# RNA regulatory networks diversified through curvature of the PUF protein scaffold

**DOI:** 10.1038/ncomms9213

**Published:** 2015-09-14

**Authors:** Daniel Wilinski, Chen Qiu, Christopher P. Lapointe, Markus Nevil, Zachary T. Campbell, Traci M. Tanaka Hall, Marvin Wickens

**Affiliations:** 1 Department of Biochemistry, University of Wisconsin, Madison, Wisconsin 53706, USA; 2 Epigenetics and Stem Cell Biology Laboratory, National Institute of Environmental Health Sciences, National Institutes of Health, Research Triangle Park, North Carolina 27709, USA

## Abstract

Proteins bind and control mRNAs, directing their localization, translation and stability. Members of the PUF family of RNA-binding proteins control multiple mRNAs in a single cell, and play key roles in development, stem cell maintenance and memory formation. Here we identified the mRNA targets of a *S. cerevisiae* PUF protein, Puf5p, by ultraviolet-crosslinking-affinity purification and high-throughput sequencing (HITS-CLIP). The binding sites recognized by Puf5p are diverse, with variable spacer lengths between two specific sequences. Each length of site correlates with a distinct biological function. Crystal structures of Puf5p–RNA complexes reveal that the protein scaffold presents an exceptionally flat and extended interaction surface relative to other PUF proteins. In complexes with RNAs of different lengths, the protein is unchanged. A single PUF protein repeat is sufficient to induce broadening of specificity. Changes in protein architecture, such as alterations in curvature, may lead to evolution of mRNA regulatory networks.

## Abstract (web summary)

Members of the PUF family of RNA-binding proteins bind multiple mRNAs *in vivo*. Here the authors show that the *S. cerevisiae* Puf5p binds targets of varying lengths that correlate with biological functions. The RNA-binding sites adopt different structures to adapt to a fixed protein scaffold.

## Graphical abstract

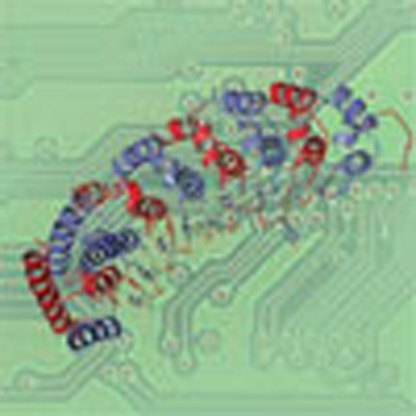

RNA-binding proteins control an messenger RNA (mRNA)’s life, including its translation, movement and destruction. These events underlie diverse biological processes, ranging from early development to memory formation. Regulatory proteins bind simultaneously to short RNA sequences, typically in 3′-untranslated regions (3′UTRs), and to protein effectors that determine the RNA’s fate. The RNA-binding specificities of the proteins determine which mRNAs are controlled, while effectors determine the outcomes.

RNA regulatory networks, in which a single RNA-binding protein controls multiple mRNAs, are widespread^1^. For example, cytoplasmic polyadenylation element-binding protein, an RNA recognition motif-containing protein, binds to and regulates many mRNAs that participate in the regulation of embryonic cell cycles^2^, while Nova co-regulates multiple mRNAs with roles in alternative polyadenylation and splicing^3^. As a result, RNA-binding proteins integrate post-transcriptional controls, as DNA-binding proteins coordinate transcriptional regulation. To understand RNA regulatory circuits in molecular terms, we need to know which mRNAs are controlled, how they are recognized, and how the networks change during evolution.

PUF proteins are exemplary mRNA regulators^4^,^5^. They bind to the 3′UTRs of many mRNAs and do so through single-stranded RNA-binding elements. For example, Puf3p of yeast binds nuclear-encoded mRNAs with roles in the mitochondria^6^. Similarly, PUF proteins in *Caenorhabditis elegans, Drosophila* and humans control an overlapping battery of mRNAs with established roles in stem cells^7^. The RNA-binding specificities of most PUF proteins are defined in part by three amino acids (tripartite recognition motifs, TRMs) in each of eight tandemly reiterated PUF repeats^8^. TRMs, annotated as XY–Z, recognize specific bases through edge-on (residues X and Y) and stacking interactions (residue Z)^9^. Specificity also can be achieved through the requirement for a base that does not contact the protein, but is solvent-exposed^8^,^9^. RNA immunoprecipitation and microarray (RIP-chip) studies suggested that *Saccharomyces cerevisiae* Puf5p binds ∼200 mRNAs that contain 10-nt-long binding elements^6^. Genetic analysis has implicated Puf5p in multiple cellular functions, including lifespan^10^, cell wall integrity^11^, chromatin structure^10^ and mating type switching^12^, consistent with the view that it participates in the control of diverse groups of mRNAs.

In this work, we used ultraviolet-crosslinking and high-throughput sequencing to define ∼1,000 high-confidence RNA targets of Puf5p *in vivo*. These targets possess unexpected diversity of binding element lengths, with the same RNA sequence features at the two ends but varying numbers of nucleotides in between. The lengths of sites correlate with the biological functions of the targets. The crystal structures of Puf5p–RNA complexes revealed that the RNAs assume altered conformations to accommodate a fixed protein architecture. The plasticity in binding element length is driven by the flattened curvature of the PUF protein scaffold. The findings suggest ways in which alterations in protein curvature result in new specificities and enable the evolution of new RNA networks.

## Results

### Identification of Puf5p RNA targets

**Figure 1:**
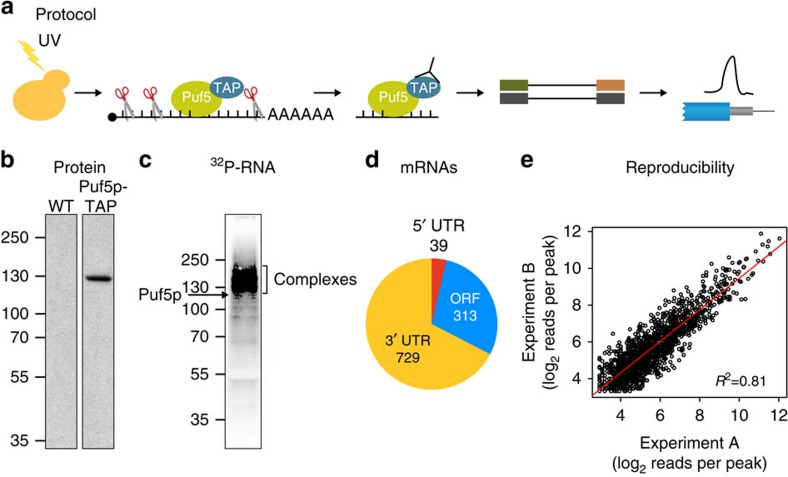
Puf5p HITS-CLIP. (**a**) Summary of HITS-CLIP protocol. *S. cerevisiae* cells were isolated and ultraviolet irradiated (254-nm wavelength). Cell lysate was subjected to gentle RNase A digestion and then TAP-tagged Puf5p was affinity purified sequentially with calmodulin and IgG resins. RNA adaptors were ligated to RNA fragments. Libraries were PCR amplified and high-throughput sequenced. (**b**) Western blot of wild type (WT) and epitope-tagged Puf5p. (**c**) Autoradiogram of ^32^P-labelled RNA crosslinked to Puf5p. Complexes migrate higher than protein alone. (**d**) Pie chart of Puf5p CLIP peaks found in mRNA regions. (**e**) Reproducibility of peaks from each biological replicate. Normalized log_2_ reads/peak for the two experiments are plotted.

Using *in vivo* ultraviolet-crosslinking and high-throughput sequencing (HITS-CLIP^13^,^14^), we identified >1,000 mRNAs to which Puf5p binds in *S. cerevisiae*, representing 16% of the yeast transcriptome (Fig. 1a). The strain analysed contained a *PUF5* gene fused to a tandem affinity purification (TAP) tag. The tagged gene was integrated by homologous recombination into the *PUF5* locus. Cells were irradiated during mid-log phase (Fig. 1a). After lysis and mild RNase treatment (Fig. 1a), Puf5p was stringently purified through tandem affinity steps (Fig. 1a) and SDS–polyacrylamide gel electrophoresis. The purification of crosslinked complexes was effective, as evidenced by western blotting (Fig. 1b). Complexes whose RNA components had been ^32^P-end labelled exhibited heterogenous, slower mobilities than Puf5p alone (Fig. 1c). To identify RNAs bound to Puf5p, adaptors were ligated, the protein digested, and the RNAs converted to complementary DNAs that were analysed by high-throughput sequencing (Fig. 1a). The adaptors contained random bar codes, so that PCR duplication events could be discarded.

On aligning the sequence reads to the yeast genome, we found that the majority of peaks were within 3′UTRs of mRNAs, and that the set of target mRNAs were distinct from, but overlapped, those of other yeast PUF proteins. We obtained 16,300,145 and 11,100,468 reads from two biological replicates. Of these, 616,401 and 491,532 (6 and 7%) mapped to unique locations in the *S*. *cerevisiae* genome, after filtering by quality score and removing PCR duplicates (Fig. 1a; Supplementary Fig. 1a). The functional enrichment of targets detected was only minimally affected by changing the filtering methods we used (Supplementary Fig. 1b). A total of 1,439 peaks were identified, representing a total of 1,190 RNAs (Supplementary Data 1). Of these, 1,043 (88%) mapped to mRNAs. The remaining 12% mapped to non-coding RNAs, including small nucleolar RNA, small nuclear RNA, non-coding RNA and transfer RNAs (Supplementary Fig. 2). Of the peaks in mRNAs, most resided in 3′UTRs (68%), but a fraction mapped to open reading frames (ORFs; 28%) or 5′UTRs (4%; Fig. 1d; see Supplementary Data 1 for complete gene list). Approximately 7% of Puf4p targets defined by RIP-chip overlap with those of Puf5p (Supplementary Fig. 3b). The overlap with Puf3p targets is 30% by CLIP analysis and 2% by RIP-chip (Supplementary Fig. 3c,d). The 1,043 Puf5p targets represent 16% of yeast mRNAs, a fivefold increase relative to the 206 targets detected in earlier RIP-chip studies^6^ (Supplementary Fig. 3a; see the Discussion section). We conclude that Puf5p is a broad regulator of a distinct set of mRNAs in *S. cerevisiae.*

### Puf5p-binding elements range in length from 8 to 12 nt

**Figure 2:**
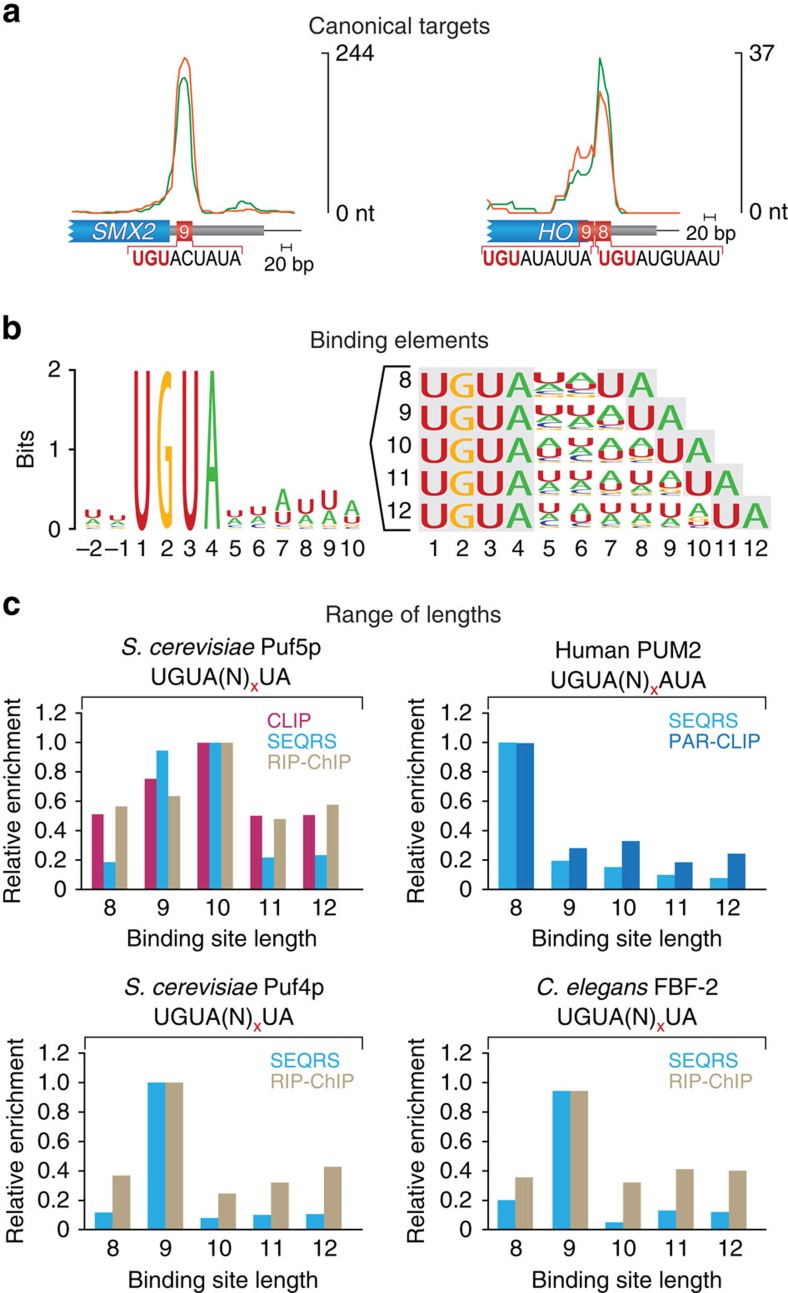
Puf5p binding element exhibits flexibility. (**a**) Puf5p interaction peaks map to previously characterized Puf5p binding sites. Orange and green lines represent reads mapped for each replicate. *SMX2* has a peak over the 9-nt-binding element. *HO* has a broad peak over two binding elements: a 9 nt lower affinity site and a 8/10 nt higher affinity site. (**b**) Binding elements identified in high-confidence Puf5p target mRNAs. The MEME-derived logo is shown on the left, which was deconvoluted into five binding elements of 8–12 nt in length. (**c**) Distribution of binding element lengths for four PUF proteins representing three species. Results from CLIP (red), SEQRS^24^ (light blue), RIP-chip^6^,^7^ (green) and PAR-CLIP^14^ (dark blue) experiments are compared, where available, and shown as enrichment relative to the predominant length for each protein, which is set to 1. The consensus RNA sequence element for each protein is shown, where N is A, C, G or U.

We developed stringent criteria to select a set of 1,043 high-confidence targets that we used to identify RNA sequence elements bound by Puf5p. We first defined significant peaks as an enrichment of independent reads in a specific genic region (modified false discovery rate (modFDR)<0.01)^15^ (Supplementary Fig. 1a). To identify high-confidence targets, we required that a peak contain 1-nt or 2-nt deletions in multiple reads (a strong indicator that these RNAs had been crosslinked to Puf5p^16^) and a minimum of 10 reads per peak. In addition, these criteria had to be satisfied in both biological replicates (Supplementary Fig. 1a). Normalized peak heights at specific loci were reproducible between the biological replicates (Pearson correlation coefficient 0.90; Fig. 1e). Previous studies validated several putative targets of Puf5p by showing they were regulated by that protein *in vivo*^17^,^18^. Among the best characterized are *SMX2* and *HO* mRNAs^19^,^20^, which are used here as examples (Fig. 2a). With both mRNAs, peaks lay over the previously characterized binding elements in their 3′UTRs (Fig. 2a).

We identified five classes of Puf5p-binding elements ranging from 8 to 12 nt, each comprising a 5′-UGUA tetranucleotide sequence and a 3′-UA with a variable length spacer region in between (Fig. 2b). We performed an unbiased search of the complete set of high-confidence targets for over-represented sequences in peaks using multiple em for motif elicitation (MEME)^21^. The position weight matrix we obtained consists of a 5′-UGUA tetranucleotide sequence followed by a degenerate 3′-end (Fig. 2b). However, we could de-convolute the complete set of 5′-UGUA-containing sequences into five classes of binding elements, ranging in length from 8 to 12 nt beginning at the 5′-UGUA, each with a 3′-terminal UA sequence (Fig. 2b). Seventy-one per cent of the 1,043 targets, and 66% of the total number of peaks (1,439), contained at least one Puf5p-binding element. Peaks without enriched sequences may reflect contacts that were less sequence-specific or mediated by interactions between Puf5p and RNA-bound factors. The sequences between UGUA and UA display little difference compared with the background nucleotide frequencies surrounding the sites (Supplementary Fig. 4), though adenosine was modestly enriched 1- or 2-nt upstream of the 3′-terminal UA in 9- and 10-nt elements (Supplementary Fig. 4a), and guanosines were uncommon (Supplementary Fig. 4c).

The breadth of binding element lengths associated with Puf5p is unusual among PUF proteins (Fig. 2c). For example, three other PUF proteins—human PUM2 (ref. 14), *S. cerevisiae* Puf4p^6^ and *C. elegans* FBF-2 (ref. 7)—show a single dominant length of site *in vivo*, measured either by CLIP methods^22^ or inferred from RIP-chip^23^ (Fig. 2c). Essentially the same behaviour was observed for each protein *in vitro*^24^. To examine the sequence preferences of Puf5p *in vitro*, the purified protein was incubated with an RNA library in which 20 consecutive nucleotides had been randomized, generating a theoretical complexity of four^20^ RNA sequences^24^. Bound RNAs were eluted, and the process repeated five times. The RNAs were analysed by high-throughput sequencing. In this method, termed SEQRS, the number of reads obtained is a proxy for affinities measured *in vitro*^24^. Re-analysis of data obtained with Puf5p^24^ revealed that the number of reads for each site length yielded a pattern similar to that seen in HITS-CLIP, in that 9- and 10-nt sites were the most abundant (Fig. 2c). Eight, 11 and 12-nt sites were less prevalent in SEQRS than *in vivo*, but above background *in vitro*. Indeed, for each protein analysed, we observed greater binding for sub-optimal lengths in the cell than *in vitro* (Fig. 2c). Many factors affect binding *in vivo*, including protein–protein interactions and RNA accessibility.

### Site length correlates with biological function

**Figure 3:**
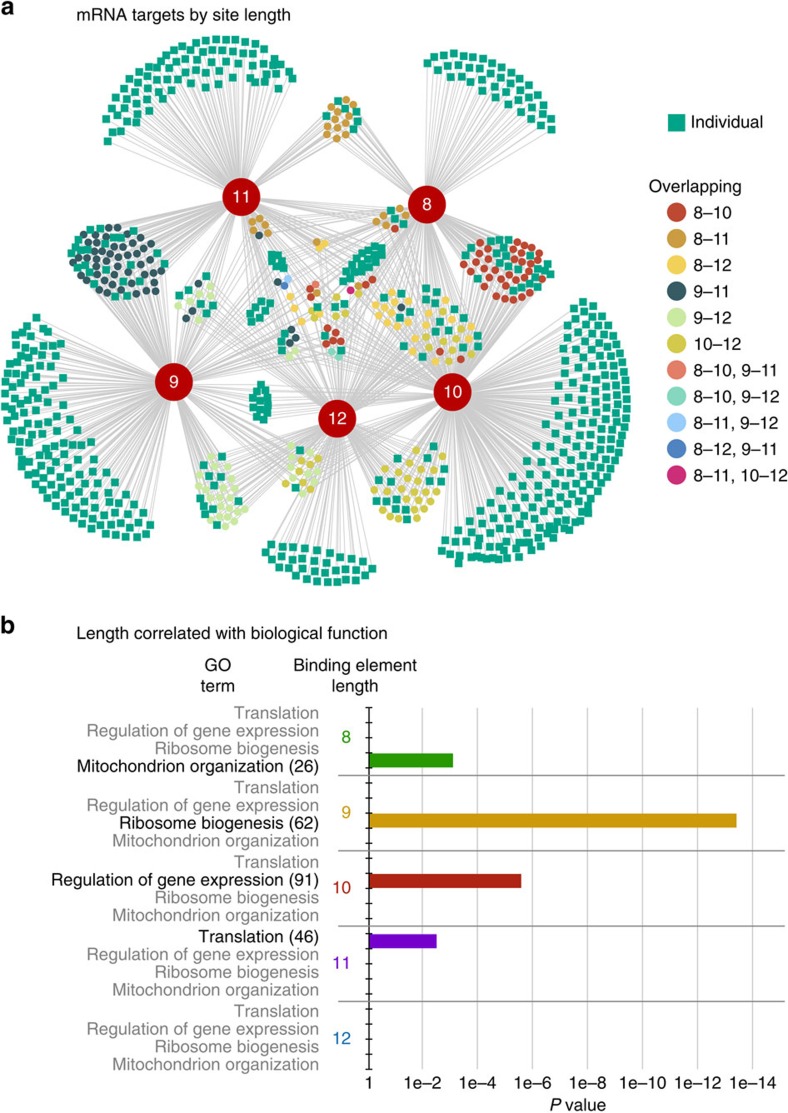
Binding element lengths correlate with biological functions. (**a**) Network representation of Puf5p mRNA targets visualized in Cytoscape 3.0.1 (ref. 47). Binding elements were defined as UGUN_(x)_UA within 25 bp of the peak summit. Large red hubs indicate the length of the binding element. Each node (small circle or square) represents one HITS-CLIP peak in an mRNA. Green square nodes represent mRNAs containing an individual binding element of either 8, 9, 10, 11 or 12 nt; nodes with only one binding element (edge) are placed at the outer periphery of the diagram. Green square nodes with two lines indicate that the HITS-CLIP peak contained two non-overlapping binding elements. Most binding elements were unambiguously of a single length. In a minority of elements, two different lengths of binding elements co-reside in a single sequence. Circles represent these mRNAs with ‘overlapping’ binding elements: for example, ‘8–10’ means a single site of the sequence UGUNNNUAUA, which possesses both 8- and 10-nt elements depending on the 3′-UA used, and either sequence may be used *in vivo*, and ‘8–10, 9–11’ means that two distinct overlapping sites are present under the peak. The key to the right is a colour code for each combination of overlapping binding element lengths (nt). The numbers of mRNAs containing overlapping binding elements are provided in Supplementary Table 1. (**b**) Gene ontology term enrichment for mRNAs belonging to each length of binding element using SGD YeastMine^39^. GO terms that are significantly over-represented in the gene list are bolded for each binding element length. Numbers of genes in the most enriched GO terms are in parentheses.

The majority of the sites bound by Puf5p consist of individual elements, in which only a single site of unambiguous length is present in the CLIP peak (Fig. 3a; Supplementary Table 1; Supplementary Data 2). Gene ontology (GO) analysis of the Puf5p targets suggests that each binding element length found in mRNAs correlates with a distinct biological role (Fig. 3b; Supplementary Table 2). Surprisingly, when we analysed mRNAs with different binding site lengths separately, RNAs with 8-nt binding elements were over-represented for mitochondrion organization (hypergeometric distribution test—correction Holm–Bonferroni, *P* value 9.5e−4); 9-nt sites for ribosome biogenesis (hypergeometric distribution test—correction Holm–Bonferroni, *P* value 3.6e−14); and 10-nt sites for regulation of gene expression (hypergeometric distribution test—correction Holm–Bonferroni, *P* value 2.9 e−6), 11-nt sites for translation (hypergeometric distribution test—correction Holm–Bonferroni, *P* value 3.6e−3) (Fig. 3b). A total of 12-nt sites did not correlate with a specific GO term. GO analysis of the mRNAs with binding elements in ORFs or 5′-UTRs, revealed that only 10-nt-binding elements in ORFs were associated with a GO term, positive regulation of pseudohyphal growth (hypergeometric distribution test—correction Holm–Bonferroni, *P* value 8.1E−3).

The 8-nt Puf5p elements lie in a subset of mRNAs that also were bound to Puf3p in PAR-CLIP experiments^25^. Puf3p associated with ∼1,000 nuclear-encoded mRNAs with mitochondrial functions^25^. Even if the criteria selecting high-confidence Puf5p targets are relaxed—not filtering for gapped reads—a very similar enrichment emerges (Supplementary Fig. 1b). Similarly, 22% of the 9-nt Puf5p elements lie in mRNAs that bind Puf4p and are enriched for genes with ribosome assembly and nucleolar functions. Of the Puf5p targets with GO annotations mitochondrion organization or ribosome biogenesis, 46 and 31% are Puf3p or Puf4p targets, respectively^6^. We suggest that the restricted specificities of Puf3p and Puf4p for 8- and 9-nt sites, respectively, underlie the correlations between Puf5p length of binding sites and their biological functions. In particular, we suggest that the broadened specificity of Puf5p enabled its recruitment to pre-existing RNA regulatory circuits in the *S. cerevisiae* lineage (see the Discussion section).

### Alternate RNA conformations adapt to fixed protein scaffold

**Figure 4:**
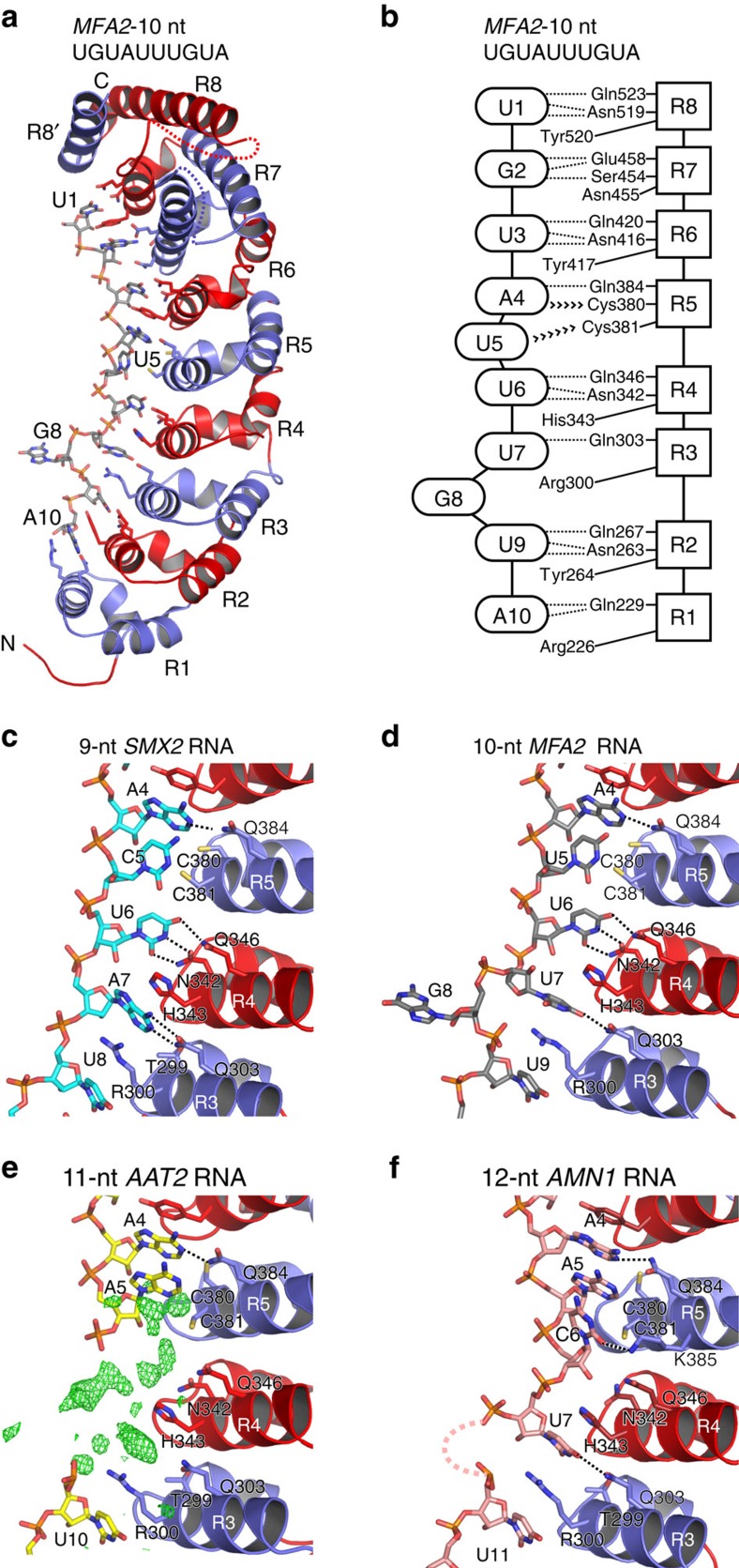
Crystal structures of Puf5p in complex with representative 9–12 nt target mRNAs. (**a**) Crystal structure of Puf5p in complex with 10-nt *MFA2* RNA. Puf5p is shown as a ribbon diagram with PUM repeats coloured alternately blue and red. Two disordered loops are indicated with dotted lines. RNA-interacting residues and *MFA2* RNA are shown as stick models with atoms coloured by element (carbon, grey; nitrogen, blue; oxygen, red; sulfur, yellow; phosphorus, orange). (**b**) Schematic diagram of interactions between Puf5p repeats (rectangles) and *MFA2* RNA bases (ovals). Hydrogen bonds are indicated by dotted lines and van der Waals contacts are indicated by >>>>. (**c**–**f**) Interactions between repeats 3–5 of Puf5p and 9-nt *SMX2* RNA (**c**), 10-nt *MFA2* RNA (**d**), 11-nt *AAT2* RNA (**e**) or 12-nt *AMN1* RNA (**f**). Hydrogen bonds are indicated with dotted lines. Discontinuous *F*_o_−*F*_c_ electron density (contoured at 3*σ*) following base A5 in the 11-nt *AAT2* RNA is shown in **e**.

To understand how Puf5p accommodates a wide range of target site lengths, we determined crystal structures of the Puf5p RNA-binding domain bound to RNAs of 9–12 nt (Fig. 4; Table 1), including the recognition sequences in *SMX2* (9 nt, 2.35-Å resolution), *MFA2* (10 nt, 2.15-Å resolution), *AAT2* (11 nt, 2.5-Å resolution) and *AMN1* (12 nt, 2.8-Å resolution) mRNAs (sites of 8 nt did not crystallize). Each RNA corresponded to a high-confidence 3′UTR-binding element in HITS-CLIP (Fig. 2a; Supplementary Figs 5,6). The RNAs bound with affinities that corresponded to our structural observations, in that higher affinity RNA-binding sites correlated with larger numbers of protein:RNA contacts (Fig. 4; Supplementary Fig. 7; Supplementary Table 3).

Despite the varying lengths and sequences of the RNA-binding sites, the overall conformation of Puf5p was unchanged in the four crystal structures (root mean squared deviation<0.7 Å over all Cα atoms or <1.1 Å over all protein atoms). The protein scaffold comprises eight α-helical repeats flanked by a short N-terminal sequence and a C-terminal helix (R8′) (Fig. 4a). The C-terminal repeats 5–8 bound the 5′-UGUA RNA sequence, while repeats 1 and 2 bound the UA-3′ element (Fig. 4a,b). Repeats 3 and 4 lie opposite the variable central regions of the RNAs (Fig. 4c–f). While the overall architecture of Puf5p resembles that of other PUF proteins, Puf5p’s repeats are more irregular in length and structure than seen in human PUM1 and *S. cerevisiae* Puf3p and Puf4p (Supplementary Fig. 8a). For example, repeats 7 and 8 in Puf5p are unusually long (64 and 72 residues versus 36 in a typical repeat) with extended α2 and α3 helices and inter-helix loops. The positions of the α3 helices relative to the α1 and α2 helices are also more varied in Puf5p than Puf3p or Puf4p (Supplementary Fig. 8a).

Since the curvature of the Puf5p scaffold is fixed, RNAs of different lengths adopt different conformations, as described below. Recognition of the 5′-UGUA and 3′-UA elements by repeats 5–8 and 1–2, respectively, are identical in all structures. Differences in RNA conformation and recognition are found opposite the central repeats 3 and 4.

Puf5p binds to the 9-nt *SMX2* RNA site by recognizing all but the central fifth base. (Fig. 4c). Bases 1–4 and 6–9 are each recognized by a PUF repeat (Supplementary Fig. 5a). However, the fifth base, C5, lies in an atypical conformation, in which the plane of the base is parallel to the axis of the protein, within van der Waals bonding distance of the side chain of Cys381 in repeat 5 (Fig. 4c). The ribose rings of C5 and U6 adopt C2′-endo conformations to accommodate positioning base C5. We refer to this conformation as ‘5-parallel’.

Puf5p binds the 10-nt *MFA2* site similarly to the 9-nt *SMX2* site, but an additional base is accommodated by turning the eighth base away from the RNA-binding surface opposite repeat 3, which we refer to as ‘8-flipped’ (Fig. 4d). The positions for all but the eighth base overlap with the 9-nt *SMX2* RNA, and the protein:RNA recognition pattern is similar, though the seventh base is a uracil in *MFA2* and an adenine in *SMX2* (Fig. 4b; Supplementary Fig. 5a).

Puf5p appears to recognize only the 5′-UGUA conserved element and two additional 3′ bases of the 11-nt *AAT2* site (Fig. 4e; Supplementary Fig. 5b), consistent with weaker binding of Puf5p to this site than 9- or 10-nt sites (12- or 2-fold weaker binding, respectively, Supplementary Table 3). A 2.5-Å crystal structure of Puf5p:*AAT2* reveals electron density for bases 1–5 and for two 3′ bases bound to Puf5p repeats 1 and 2. In contrast to the parallel orientation of base 5 in 9 and 10-nt sites, base A5 of *AAT2* stacks directly with base A4 and forms a van der Waals contact with the side chain of Cys381 in Puf5p repeat 5 (Fig. 4e). We refer to this conformation as ‘5-stacked’. Using the consensus sequences as a guide, we modelled the 3′ bases as the conserved U10 and A11 bases and did not model bases 6–9. However, alternate conformations of the RNA are possible, including a conformation similar to that of the 12-nt *AMN1* site.

Puf5p binds to the longer 12-nt *AMN1* site with a distinct RNA conformation. Unlike the conformations of the shorter length binding sites, bases A4, A5 and C6 stack directly with each other opposite repeat 5. We refer to this conformation as ‘Triple-stacked’. Residues in repeat 5 (Cys381 and Lys385) contact bases A5 and C6 (Fig. 4f). Puf5p repeat 4 does not interact with an RNA base using its edge-interacting residues, but base U7 is bound to repeat 3 (Fig. 4f). Electron density was observed for bases 1–7 and two 3′ bases bound to Puf5p repeats 1 and 2. We modelled the 3′ bases as the conserved U11 and A12 bases, as we did for the 11-nt *AAT2* site, and bases 8–10 were not included in the model.

### Curvature as a determinant of specificity

The flatter RNA-binding surface of Puf5p contributes to its specificity by creating a more extended RNA-binding surface. Puf5p possesses the least curved RNA-binding surface observed among PUF proteins to date (Supplementary Fig. 8b) and binds to the longest RNA target sequences identified thus far. Puf3p preferentially binds 8-nt sites and exhibits the greatest curvature among the yeast PUFs (Supplementary Fig. 8b); this reflects the regular spacing of RNA-binding helices, which matches the spacing of bases in an extended RNA chain^26^. Puf4p, which binds 9-nt-binding sites, is intermediate in curvature, between Puf3p and Puf5p (Supplementary Fig. 8b).

Extension of the Puf5p RNA-binding surface is produced by the structural arrangements in repeats 4 and 5 and corresponds to the variability in Puf5p target sequence length relative to other PUF proteins. The largest repeat-to-repeat angle in Puf5p is centred about repeat 5 (Supplementary Fig. 8c). Repeat 5 also lacks a large side chain capable of stacking with RNA bases and lies opposite several of the atypical RNA conformations (5-parallel, 5-stacked and triple-stacked). The flatness combined with a protein surface lacking specificity allows ‘extra’ RNA nucleotides, needed to span the distance between repeats with base specificity, to assume different conformations. These extra nucleotides may not contact the protein, but instead stack with one another or lie parallel to the RNA-binding surface.

### Evolution of binding specificity across Ascomycota

**Figure 5:**
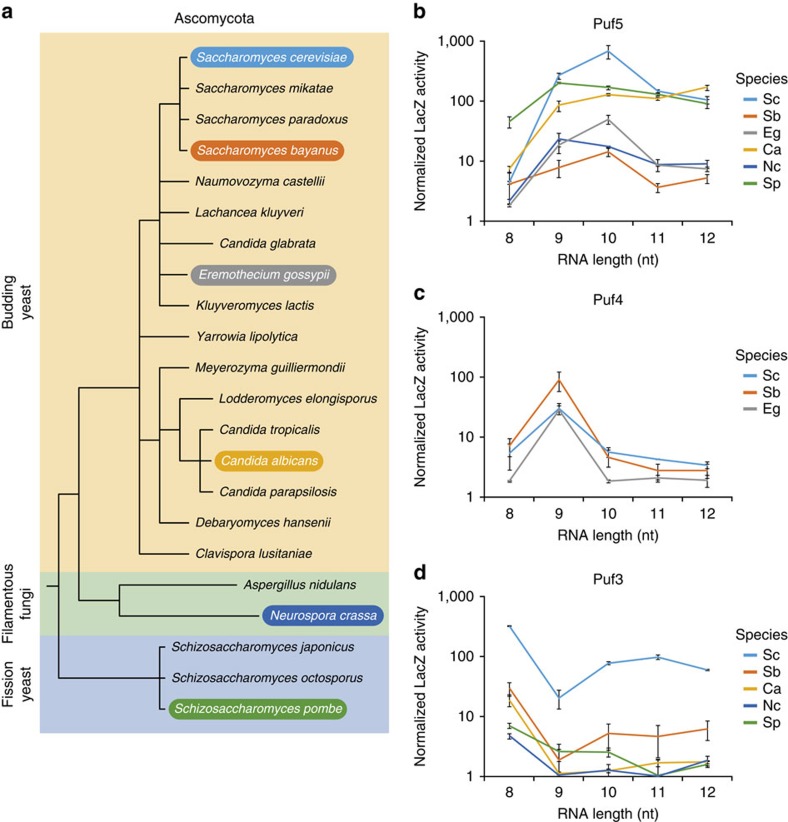
Binding element preference across phylum Ascomycota. (**a**) Phylogeny of Ascomycota fungi. Each subphylum is indicated: budding yeast—yellow; filamentous fungi—green; and the fission yeast—blue. Species used for RNA-binding studies below are highlighted. (**b**–**d**) RNA-binding element length preferences for Puf5p, Puf4p and Puf3p orthologues. RNA binding was assayed using the yeast three-hybrid system^48^. RNAs tested were 8 (5′- UGUAAAUA -3′), 9 (5′- UGUAAAAUA -3′), 10 (5′- UGUAAAAAUA -3′), 11 (5′- UGUAAAAAAUA -3′) or 12 (5′- UGUAAAAAAAUA -3′) nucleotides in length. Raw luminescence values per cell for each biological replicate (*n*=3) were averaged then normalized to controls where the 5′-UGU sequence was mutated to ACA. Error bars represent s.d. The full set of six species could not be tested for all five binding element lengths. *N. crassa* does not have a Puf4 ortholog; *C. albicans* and *S. pombe* Puf4p failed to specifically bind any RNA; *E. gossypii* Puf4p clones could not be obtained.

To examine the evolution of the broad specificity of Puf5p, we probed the RNA-binding preferences of Puf5p proteins from representative species across phylum Ascomycota. This group includes the budding yeasts, filamentous fungi and fission yeasts (Fig. 5a). We used the yeast three-hybrid assay to measure the affinities of Puf5p orthologues from six different species—*S. cerevisiae*, *Saccharomyces bayanus*, *Eremothecium gossypii*, *Candida albicans*, *Neurospora crassa* and *Schizosaccharomyces pombe* (Fig. 5a). These proteins were identified as orthologues using SYNERGY, which relies on the species tree, sequence similarity and synteny^27^. Their binding preferences versus length of site were evaluated using a set of RNAs 8–12 nt in length, conforming to the sequence UGUA(A)_2–5_UA using the yeast three-hybrid system^28^. All the RNAs thus maintained the 5′-UGUA and 3′-UA critical for *S. cerevisiae* Puf5p interaction and contained a single 3′-UA element to define the target length unambiguously. In the three-hybrid assay, the level of expression of a reporter gene (LacZ) is a proxy for the affinity of the interaction^29^.

Puf5p proteins across Ascomycota exhibited broad binding specificities. Puf5 proteins bound similarly to sites of 9, 10, 11 and 12 nt (Fig. 5b). The more restricted specificities of Puf4p for 9-nt sites (Fig. 5c), and Puf3p for 8-nt sites (Fig. 5d), also were conserved across the entire phylum, with the exception of *S. pombe* Puf3. This protein bound a broad range of site lengths, unlike its orthologues in other species that showed preference for 8-nt sites.

**Figure 6:**
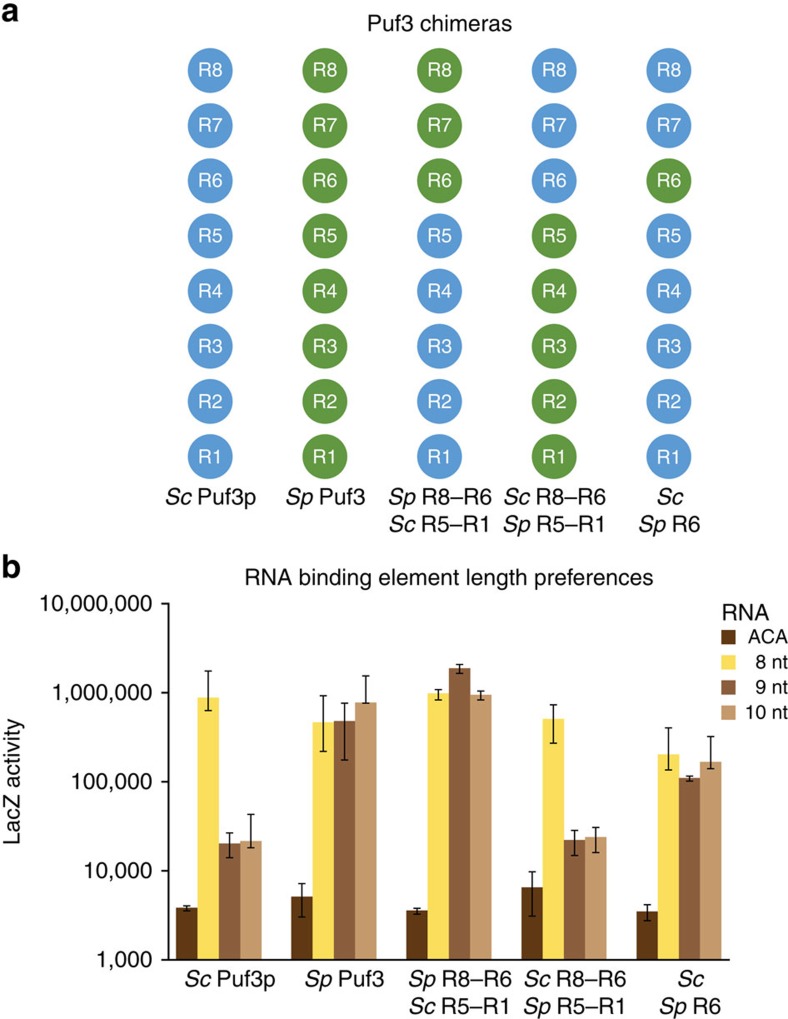
Evolution of Puf3p broadened RNA specificity. (**a**) Schematic representations of chimeras tested in **b**. PUM repeats are represented by circles: *S. cerevisiae*, blue; *S. pombe*, green. (**b**) Broadened *S. pombe* Puf3 specificity is linked to repeat 6. RNA-binding element length preferences for the chimeric proteins were assayed using the yeast three-hybrid system with 8, 9 or 10 nt RNAs as in Fig. 5. Raw luminescence values per cell for each biological replicate (*n*=3) were averaged then normalized to an acaAAAUA mutant negative control, which depresses binding >100-fold^49^. Error bars represent s.d.

The broadened specificity of *S. pombe* Puf3 appears to have arisen exclusively in the fission yeast lineage, which enabled us to probe how that broadening arose during evolution. We reasoned that the broadening was not due to the identity of the RNA-interacting TRMs, as the residues are identical among all the Puf3p orthologues (with the exception of repeat 3 in *N. crassa*, with a Gln to Arg substitution). To identify the key regions of the proteins that confer specificity, we prepared chimeras in which segments of the *S. cerevisiae* and *S. pombe* proteins were exchanged (Fig. 6a). The specificity profile—broad or narrow—was conferred by PUF repeats 6–8. A chimeric protein possessing repeats 6–8 from *S. pombe* exhibited broad specificity, while a chimera with repeats 6–8 of the *S. cerevisiae* protein had narrow specificity (Fig. 6a,b). The protein sequences in repeat 6 contain a divergent region among Puf3p orthologues (Supplementary Fig. 9). Indeed, substitution of *S. pombe* repeat 6 alone into an *S. cerevisiae* scaffold was sufficient to confer the broad specificity profile (Fig. 6b).

## Discussion

Puf5p is a broad regulator of RNAs in *S. cerevisiae*, binding to >1,000 RNA targets, constituting ∼16% of the transcriptome. A total of 71% of these targets possess recognizable binding elements beginning with a 5′-UGU sequence, which range in length from 8 to 12 nt. The variations in length are accommodated by conformational adaptations of the RNA onto a fixed protein scaffold. The wide range of mRNA target site lengths is consistent with prior studies that linked Puf5p to a spectrum of functions, including cell wall integrity^11^ and chromatin structure^10^.

The biological functions of target mRNAs are correlated with the length of binding elements they possess. How does this correlation arise? We propose that the correlation is imposed by other RNA-binding proteins that recognize the same binding elements, and whose specificity is much more restricted than Puf5p (Fig. 5b–d). For example, Puf3p binds 8-nt sites that are largely in mRNAs with mitochondria-related functions, while Puf4p binds 9-nt-binding elements in mRNAs with roles in ribosomal biogenesis and assembly^6^.

Two PUF proteins that bind the same site could do so sequentially, competitively or cooperatively. Genetic studies demonstrate that Puf4p and Puf5p redundantly control the decay rate of common targets^30^. In the absence of one of the proteins, the other is sufficient. However, for other common targets, the actions of two PUF proteins may be sequential. For example, *MRPL8* mRNA is a target of both Puf5p and Puf3p, possesses a single binding element, and is localized to the mitochondrial periphery in a Puf3p-dependent manner^31^. Puf5p could exchange with Puf3p, facilitating repression (Puf5p) *en route* to localization to the mitochondria (Puf3p).

While 71% of Puf5p targets possess discernible binding elements, 29% do not. RNAs without binding elements may associate with Puf5p indirectly, perhaps through a protein to which it and Puf5p are bound. Crosslinking to RNAs without sites could also be driven by their high concentrations in specific subcellular compartments (such as P-bodies), in which proteins and RNAs are present at high concentrations, and low complexity, Q/N-rich regions present in Puf3p, Puf4p,and Puf5p proteins that could facilitate aggregation^32^.

RNAs of different lengths adopt a broad range of conformations when bound to Puf5p. The flatter, extended scaffold of Puf5p, combined with its specificity for 5′ and 3′ sequences, imposes the requirement for these RNA conformational variations and permits recognition of 8–12-nt length RNAs. The elegance of this arrangement is that very similar sets of atomic contacts between amino acids and RNA bases are maintained in the different complexes, despite the range of RNA lengths they possess. For example, 18 of the 21 edge-on contacts made between Puf3p and its RNA target are also made in Puf5p bound to a 10-nt length site. In an analogous manner, β-catenin maintains a fixed scaffold to recognize peptides from different ligands (reviewed in ref. 33). Its central α-helical Armadillo (ARM) repeats interact with conserved sequence elements in an extended peptide while N- and C-terminal ARM repeats bind elements unique to that ligand. The changes in repeat-to-repeat arrangement at the junctions between the central ARM repeats and N or C-terminal repeats seem to mark the regions with different protein-binding functions. In the same manner, changes in curvature at specific repeat junctions in PUF proteins correlate with specialization in RNA-binding specificity.

The fact that a single repeat can broaden or narrow specificity (Fig. 6) suggests that this sort of change may be common in evolution (Fig. 6b). The sixth PUF repeat of Puf3p determines whether that protein binds 8-nt sites (*S. cerevisiae*) or accommodates 8, 9 or 10-nt sites (*S. pombe*). *S. cerevisiae* repeat 6, which induces narrow specificity, contains additional residues relative to the same region of the *S. pombe* protein, which, although not near the RNA-binding residues, may alter the structure with corresponding effects on specificity (Supplementary Figs 8a and 9).

From an evolutionary perspective, the broadening of Puf5p’s specificity may have enabled new regulatory inputs into existing RNA circuits. Perhaps, the ability of Puf5p to recognize a wide array of target lengths arose after ancestral proteins (for example, Puf3p) already regulated batteries of RNAs with related functions and conserved lengths of sites. Recruitment of Puf5p to these same targets, enabled by its flatter curvature, provided new regulatory inputs and/or redundancy into that same circuit. For example, Puf5p binds regulatory kinases^34^, whose input could be brought to bear on a pre-existing circuit. Regardless, we suggest that curvature of the scaffold is critical in defining the RNAs that are controlled. Acquisition of new RNA specificities by alterations of the protein’s architecture suggests ways in which new RNA circuits are established, expanded and contracted during evolution.

## Methods

### HITS-CLIP

HITS-CLIP was performed on 8 l of *S. cerevisiae* harbouring an integrated TAP tag sequence at the C terminus of the endogenous *puf5* locus (GE YSC1178-202231131) per biological replicate. Cells were pelleted, washed with water and distributed into Petri dishes. The cells were ultraviolet irradiated with 1,000-mJ wavelength 254 light. Pelleted cells were snap frozen in liquid nitrogen and then ground with a liquid nitrogen cooled mortar and pestle. The ground material was thawed in ∼10 ml CBB (25 mM Tris (pH 7.5), 150 mM NaCl, 1 mM magnesium acetate, 1 mM imidazole, 2 mM CaCl_2_, 0.1% IGEPAL, 10 mM β-mercaptoethanol) at 25 °C. A measure of 30 μl of RQ1 DNASE (Promega, M6101) and 10 μl 1:10,000 dilution of RNAse A (Promega A7973) were added and incubated at 37 °C for 10 min. The lysate was cleared by centrifugation then incubated with 400 μl pre-washed calmodulin Sepharose beads (GE 17-0529-01) rotating at 4 °C for 1 h. Beads were washed with 10 ml CBB and then incubated in 3 ml CEB (10 mM Tris-HCl (pH 8.0), 150 mM NaCl, 1 mM magnesium acetate, 1 mM imidazole, 2 mM EGTA, 0.1% IGEPAL, 10 mM β-mercaptoethanol) for 45 min rotating at 4 °C, followed by washing with 2 ml CEB. The supernatant was incubated with 400 μl pre-washed IgG beads (Life Techonologies 11202D) rotating at 4 °C for 1 h. Following incubation, beads were washed 1 × with PNK (50 mM Tris (pH 7.5), 10 mM MgCl_2_, 0.5% IGEPAL) buffer, 3 × with high-salt buffer (15 mM Tris (pH 7.5), 5 mM EDTA, 2.5 mM EGTA, 1% Triton X-100, 1% Na-DOC, 0.1% SDS, 1 M NaCl) and then 1 × with PNK buffer.

Beads were incubated in 1 × PNK pH 6.5 buffer, 1 unit T4 PNK (NEB M0201s), 100 units RNasin (Promega N2511) and 5 units alkaline phosphatase (Promgea M1821 ) at 37 °C for 20 min shaking every 3 min for 15 s followed by washing 2 × with 1 × PNK+EGTA buffer (50 mM Tris (pH 7.5), 20 mM EGTA, 0.5% IGEPAL) and 2 × with 1X PNK buffer. Adaptor ligation was performed in 1 × T4 RNA ligase buffer (NEB), T4 RNA ligase truncated (NEB M0242L) RNasin, 1 μM pre-adenylated 3′-adaptor (5′- TGGAATTCTCGGGTGCCAAGG -3′), 10% PEG400 (Sigma, 81170) overnight at 16 °C shaking every minute for 15 s. RNAs were labelled with 5 μl P^32^-γ-ATP, 50 units T4 PNK (Promega M4101) in 1 × PNK buffer at 37 °C for 10 min shaking every 5 min for 15 s. Ten microlitres of 10 mM ATP was added to the reaction and incubated for an additional 5 min. The reaction was quenched by washing 2 × with high-salt buffer and then 2 × with 1 × PNK.

The beads were resupsened in 30 μl of 1 × LDS sample buffer (Life Technologies NP0008) including 1 μl sample reducing agent (Life Technologies NP0004). The reaction was incubated at 70 °C for 10 min and loaded in one lane of a 4–12% NuPAGE Novex Bis-Tris (Life Technologies NP0321BOX) gel. The gel was run at 150 V until the dye front ran off the gel. The complexes were then transferred to a nitrocellulose membrane (Novex LC2001) in a XCell II Blot module (Novex EI0001) at 30 V for 1 h at 4 °C. The membrane was put on a phosphor screen to visualize the Puf5p–RNA complexes. Complexes were cut out of the membrane. Nitrocellulose pieces were then incubated with 200 μl of 4 mg ml^−1^ Proteinase K (Thermo Scientific E00491) in 1 × PK buffer at 37 °C for 20 min while shaking. A measure of 200 μl of 1 × PK 7 M urea was added and then incubated at 37 °C for 20 min. RNA was phenol chloroform extracted and precipitated with ethanol:isopropanol (1:1), NaOAc and glycoblue (Life Technologies AM9516) overnight at −20 °C. The pellets were washed and resuspened in 6.9 μl water.

5′-RNA adaptor ligation was performed in 1 × T4 RNA ligase buffer, bovine serum albumin 1 μl (1 mg ml^−1^) T4 RNA ligase, 1 μl (10 μ μl; Thermo Scientific EL0021) 5′-adaptor (5′- GUUCAGAGUUCUACAGUCCGACGAUCNNNNN -3′) and incubated at 16 °C for 2 h. The reaction was quenched by phenol chloroform extraction then precipitated as above.

The pellets were washed and resuspended in 10 μl water. dNTPs (0.5 mM) and 10 uM RT primer (5′- GCCTTGGCACCCGAGAATTCCA -3′) were combined and heated to 65 °C for 5 min then cooled on ice. Reverse transcriptase buffer (1 × ), 10 mM dithiothreitol (DTT), 20 units RNasin and 200 units SuperScript II (Life Techonlogies 18064-022) were combined and incubated at 50 °C for 45 min, 55 °C for 15 min and then 90 °C for 5 min. Reverse transcriptase reactions were PCR amplified by adding 10 μl of the reaction to 15 μl GoGreen Taq (Promega M7123) and 10 μM of each primer (forward 5′- AATGATACGGCGACCACCGAGATCTACACGTTCAGAGTTCTACAGTCCGA -3′; reverse 5′- GCCTTGGCACCCGAGAATTCCA -3′). Thirty PCR cycles (95 °C for 30 s, 60 °C for 30 s and 72 °C for 30 s followed by 72 °C for 5 min) were used to amplify the libraries. PCR reactions were purified by 1% agarose TBE gel. Smears corresponding to 150–300 bp were gel purified and ethanol precipitated as above. Libraries were bar-coded (5′- CAAGCAGAAGACGGCATACGAGATXXXXXXGTGACTGGAGTTCCTTGGCACCCGAGAATTCCA -3′) using the same PCR as above for 10 cycles except replacing the reverse primer with bar-coded primer and submitted to the UW-Madison sequencing facility for sequencing on one highly multiplexed Illumnia HighSeq 2000 lane.

### Western blot

A measure of 50 μl of IgG beads were removed from CLIP samples and then incubated in 30 μl LDS sample buffer (Life Technologies NP0007). The whole reaction was run on a Novex 6% TBE gel then transferred to PDVF membrane (Millipore IPVH00010). The membrane was probed with TAP Tag Polyclonal Antibody (1:10,000; Pierce:CAB1001) primary antibody followed by goat anti-mouse secondary antibody (1:10,000; KPL:074-1506).

### Informatic pipeline

FASTQ files were uploaded to the Galaxy server^35^ and groomed (FASTQ Groomer)^36^. Adaptor sequences were then trimmed using Clip discarding sequences that contained the 5′-adaptor or were too short after 3′-adaptor clipping. The data were then filtered based on quality score using Filter FASTQ with a minimum length of 15 bases and a minimum quality score of 20. The 5′-adaptor included a 3′-random bar code that was used to remove PCR duplicates by discarding any read with a perfect duplicate.

The filtered reads were mapped to the *S. cerevisiae* genome using Bowtie2 (ref. 37; bowtie2 -x /Scgenome -q filename.fastq -S filename.sam -5 5 -N 1 -p 8). The .sam files were used to create.bam and indexed.bam files using samtools for visualization of the data in Artemis Genome browser. Peaks were defined using Pyicoteo^15^ (python pyicoclip filename.sam -f filename.pk–region Sc.bed–stranded). The .bed file required for Pyicoteo was downloaded from the Saccharomyces Genome Database (SGD)^38^. Next, the duplicate peaks were removed from the.pk file. Using the Pyicoteo defined summit, each peak was assigned to a genomic feature using the features table from the SGD. Sequences 200 bases upstream of the ORF and 300 bases downstream of the ORF were used as 5′UTRs and 3′UTRs, respectively, and then added to the SGD features table. The number of gapped reads for each peak was defined. Kurtosis was calculated for each peak using the peak profile defined by Pyicoteo. A total of 25 bases of genomic sequence flanking each peak summit was retrieved to define binding elements in two ways. MEME was used as an unbiased search and direct searches were used for known binding elements.

The biological replicates were combined into one list based on the following: (1) each peak had a summit within 10 bases in both replicates; (2) each peak contained a gapped read in both replicates; and (3) each peak had a height >10 reads (third quartile) in both replicates. Functional enrichment was performed using GO analysis. Gene lists were uploaded to YeastMine where the *P* value was calculated using the hypergeometric distribution test (whole genome as background) and multiple test corrected using Holm–Bonferroni^39^.

### Protein purification

The RNA-binding domain of yeast Puf5p (residues 201–600) was subcloned into the pSMX vector with an N-terminal His_6_-SUMO tag^40^. *Escherichia coli* cells BL21 Star (DE3) carrying the Puf5p plasmid were grown in Terrific Broth media to OD_600_=∼0.8 and then protein expression was induced with 0.4 mM isopropylthiogalactoside for 20 h at 18 °C. The cell pellet was resuspended in lysis buffer containing 20 mM Tris, pH 8.0; 0.5 M NaCl; 20 mM imidazole; 5% (v/v) glycerol and 0.1% (v/v) β-mercaptoethanol and sonicated on ice. The lysate was cleared by centrifugation and loaded onto a Ni-chelating gravity column (Thermo Scientific). His-SUMO-tagged Puf5p was eluted with a buffer containing 20 mM Tris, pH 8.0; 50 mM NaCl; 0.2 M imidazole and 1 mM DTT. Ulp1 protease was added to remove the His_6_-SUMO tag, and the protein solution was loaded onto a Hi-Trap heparin column (GE Healthcare) and eluted with a gradient from 0 to 1 M NaCl in buffer containing 20 mM Tris, pH 8.0, 1 mM DTT. The fractions containing Puf5p were pooled and concentrated by Amicon filters and loaded onto a Superdex 200 16/60 column equilibrated in 20 mM HEPES, pH 7.4; 0.15 M NaCl and 2 mM DTT. The Puf5p peak fractions were pooled and concentrated in column buffer for crystallization and RNA-binding assays.

### Protein–RNA crystallization

RNAs were purchased from Thermo Scientific. Puf5p (4 mg ml^−1^) was mixed with each of the four different RNAs at a protein:RNA molar ratio of 1:1.2 and incubated on ice for 1 h. Crystals were obtained at 20 °C by hanging drop vapour diffusion, mixing 1 μl Puf5p–RNA complex with 1 μl reservoir solution of 15–20% (w/v) PEG 3350 and 0.1 M citrate Bis-Tris propane (CBTP), pH 7.6. Microseeding was performed to grow larger single crystals. Crystals were cryo-protected in crystallization solution supplemented with 15% (v/v) glycerol and flash frozen in liquid nitrogen. For phasing, a Puf5p:*SMX* RNA complex crystal was soaked in 17.5% (w/v) PEG 3350, 0.5 M KI, 0.1 M CBTP and 15% (v/v) glycerol for 5 min and then flash frozen.

### X-ray data collection

X-ray data for structures of the 9-, 10- and 12-nt RNA complexes were collected at the SER-CAT beamline at the Advanced Photon Source, Argonne National Laboratory. Data for the 11-nt RNA complex and the iodide-soaked crystal were collected at the NIEHS in-house facility equipped with a Rigaku 007HF rotating anode generator and a Saturn 92 charge-coupled device area detector system. All data were processed using HKL2000 (ref. 41).

### Structure determination

The crystal structure of a Puf5p:*SMX2* RNA complex (space group P2_1_2_1_2) was determined by combining molecular replacement (MR) with iodide single-wavelength anomalous diffraction (SAD)) phasing. The Phenix software suite was used throughout the process of structure determination^42^. The anomalous signal of the SAD data extended only to 5.0 Å, and MR or SAD alone failed to solve the structure. A truncated Puf4p structure (PDB: 3BX2) containing repeats 4–8 (residues 684–887) was used as the MR search model. Following MR, AutoSol identified eight iodide sites with Figure of Merit (FOM) of 0.33. Running AutoBuild after MR-SAD phasing produced a model with *R*_free_=44%. The model was further improved to *R*_free_=38% using the EMBL Hamburg Auto-Rickshaw web server^43^. Electron density for the 9-nt RNA was clearly visible. Iterative cycles of manual model building in Coot^44^ and refinement with Phenix led to the final model with *R*_free_=28% (Table 1).

Crystals of the 10-, 11- and 12-nt RNA complexes and some crystals of the 9-nt *SMX2* RNA complex belonged to space group P6_1_22, although all crystals were grown in the same conditions as the P2_1_2_1_2 *SMX2* crystals. These structures were determined by MR using the Puf5p coordinates from the initial Puf5p:*SMX2* structure as the search model. Data and refinement statistics are shown in Table 1. All models show good geometry according to MolProbity^45^: 95–98% of the residues are in favoured regions of the Ramachandran plot, and there are no outliers.

### Electrophoretic mobility shift assays

RNA oligonucleotides were radiolabelled using ^32^P-γ-ATP and T4 polynucleotide kinase (New England Biolabs) following the manufacturer’s instructions. Serially diluted Puf5p was mixed with 100 pM labelled RNA in buffer containing 10 mM HEPES, pH 7.4; 50 mM NaCl; 1 mM EDTA; 0.1 mg ml^−1^ bovine serum albumin; 0.01% (v/v) Tween 20 and 0.1 mg ml^−1^ yeast tRNA. After overnight, incubation at 4 °C, 4-μl loading dye (15% v/v Ficoll 400 and 0.01% bromophenol blue) was added to each 20-μl reaction before gel loading. Novex TBE gels (10%; Invitrogen) were run at 100 V at 4 °C for 30 min to resolve the samples. The gels were dried and exposed to storage phosphor screens. The screens were scanned using a Molecular Dynamics Typhoon phosphorimaging system (GE Healthcare). The band intensities were analysed using the ImageQuant. *K* values were calculated using GraphPad Prism by fitting the data assuming one-site specific binding and a Hill coefficient of 1. ∼93% of Puf5p was active, as determined using the method described in reference^46^. The reported *K*_d_ values were not adjusted.

### Yeast three-hybrid assays

Each orthologous PUF RNA-binding domain was cloned into activation domain–protein fusion plasmid, pGADT7 (ref. 28). Oligonucleotides representing each RNA sequence were ordered from Integrated DNA Technologies (IDT) and cloned into the hybrid RNA plasmid, p3HR2 (ref. 28). All experiments were conducted in the *S. cerevisiae* strain YBZ-1 (MATa, ura3-52, leu2-3, -112, his3-200, trp1-1, ade2, LYS2::(LexAop)-HIS3, URA3::(lexAop)-lacZ and LexA-MS2 MS2 coat (N55K)). Strains were lithium acetate transformed with appropriate combinations of plasmids and plated on synthetic dextrose media lacking uracil and leucine. Single colonies were selected and allowed to grow to stationary phase, then diluted and grown for about 4 h. OD_660_ for each culture was measured, then 50 μl of culture was added to 50 μl of Beta-Glo (Promgea E4720) and incubated for 1 h in the dark. Luminescence was measured by microplate reader (BioTech Synergy 4). Raw luminescence was normalized to OD_660_, and each biological replicate (*n*=3) was then averaged and s.d. was calculated.

## 

## Additional information

**Accession codes:**The Illumina FASTQ sequence files have been deposited in the Sequence Read Archive (accession numbers SRR2085177 and SRR2084791). The atomic coordinates and structure factors for the crystal structures of Puf5p in complex with *SMX2* (PDB IDs: 5BYM and 5BZV), *MFA2* (PDB ID: 5BZ1), *AAT2* (PDB ID: 5BZU) and *AMN1* (PDB ID: 5BZ5) RNAs have been deposited in the Research Collaboratory for Structural Bioinformatics Protein Data Bank.

**How to cite this article:** Wilinski, D. *et al*. RNA regulatory networks diversified through curvature of the PUF protein scaffold. *Nat. Commun.* 6:8213 doi: 10.1038/ncomms9213 (2015).

## Supplementary Material

Supplementary Figures and Supplementary Tables Supplementary Figures 1-9 and Supplementary Tables 1-3

Supplementary Data 1 List of Puf5p target RNAs. Columns indicate properties of each peak or gene information contained in the header. Each row represents a peak.

Supplementary Data 2 Cytoscape network file.

## Figures and Tables

**Table 1:** Data collection and refinement statistics.

**RNA**	**5BE9—*****SMX2*** **(A)**	**5BE9—*****SMX2*** **(B)**	**5BE10—** ***MFA2***	**5BE11—** ***AAT2***	**5BE12—** ***AMN1***
*Data collection*					
Space group	P2_1_2_1_2	P6_1_22	P6_1_22	P6_1_22	P6_1_22
Cell dimensions					
* a*, *b*, *c* (Å)	94.22, 100.79, 49.44	106.93, 106.93, 167.35	106.75, 106.75, 167.68	107.36, 107.36, 167.23	105.85, 105.85, 168.40
α, β, γ (°)	90.0, 90.0, 90.0	90.0, 90.0, 120.0	90.0, 90.0, 120.0	90.0, 90.0, 120.0	90.0, 90.0, 120.0
Resolution (Å)	50–2.70 (2.75–2.70)	50–2.35 (2.39–2.35)	50–2.15 (2.19–2.15)	50.0–2.50 (2.54–2.50)	50.0–2.80 (2.85–2.80)
*R*_sym_	0.049 (0.239)	0.098 (0.404)	0.105 (0.565)	0.051 (0.439)	0.094 (0.568)
*I*/σ*I*	38.0 (6.2)	22.9 (3.8)	22.9 (2.2)	18.5 (2.0)	20.0 (2.0)
Completeness (%)	95.9 (73.9)	93.0 (83.2)	99.3 (94.6)	98.2 (96.9)	93.7 (91.7)
Redundancy	6.8 (5.6)	7.3 (4.9)	10.8 (6.3)	5.0 (5.0)	6.9 (4.2)
					
*Refinement*					
Resolution (Å)	42.7–2.70	45.0–2.35	40.5–2.15	32.4–2.50	38.5–2.80
No. of reflections	12,790	22,610	31,174	19,884	13,471
*R*_work_/*R*_free_	24.1/28.5	19.2/23.6	17.9/21.7	23.4/27.4	20.1/25.9
					
					
*No. of atoms*					
Protein	2,925	2,952	2,990	2,931	2,945
RNA	186	186	230	150	190
Water	0	145	208	57	13
					
*B-factors*					
Protein	86.0	41.8	41.4	63.4	66.4
RNA	108.3	54.6	45.8	87.0	84.3
Water	—	42.7	43.8	50.3	56.1
					
*r.m.s deviations*					
Bond lengths (Å)	0.004	0.005	0.004	0.004	0.003
Bond angles (°)	0.71	0.87	0.79	0.75	0.67
PDB	5BYM	5BZV	5BZ1	5BZU	5BZ5

r.m.s., root mean squared.

Values in parentheses are for highest-resolution shell.
