# Supplementary material for: RNA regulatory networks diversified through curvature of the PUF protein scaffold
Source: Nat Commun. 2015 Sep 14;6:8213. doi: 10.1038/ncomms9213 (PMC4570272; doi:10.1038/ncomms9213)
Supplement: Supplementary Figures and Supplementary Tables — Supplementary Figures 1-9 and Supplementary Tables 1-3 [file ncomms9213-s1.pdf]

SUPPLEMENTARY FIGURES

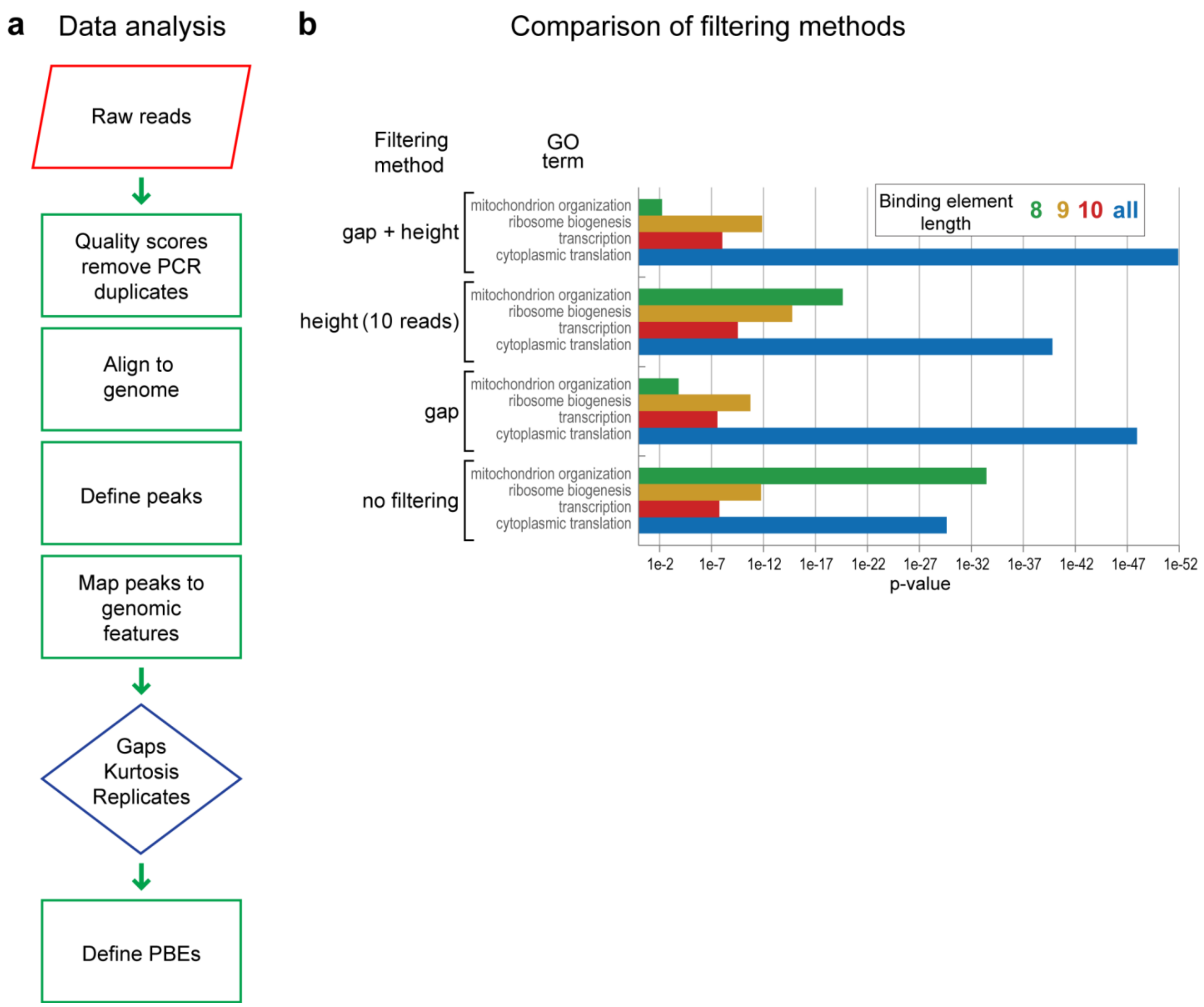

Supplementary Figure 1. HITS-CLIP data analysis pipeline. a. Flow chart of the data analysis. b. GO analysis of target RNA list using various criteria for filtering peaks using SGD YeastMine[1].

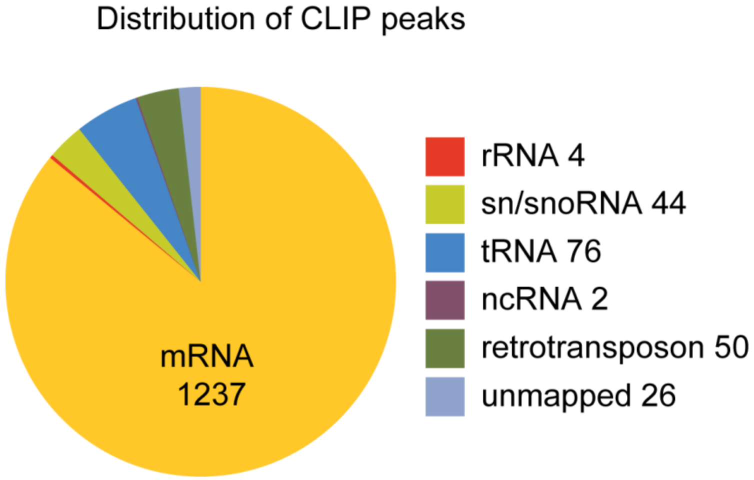

Supplementary Figure 2. Puf5p binds predominantly mRNAs. Distribution of Puf5p HITS-CLIP peaks in RNA types. Numbers of peaks in each category are indicated.

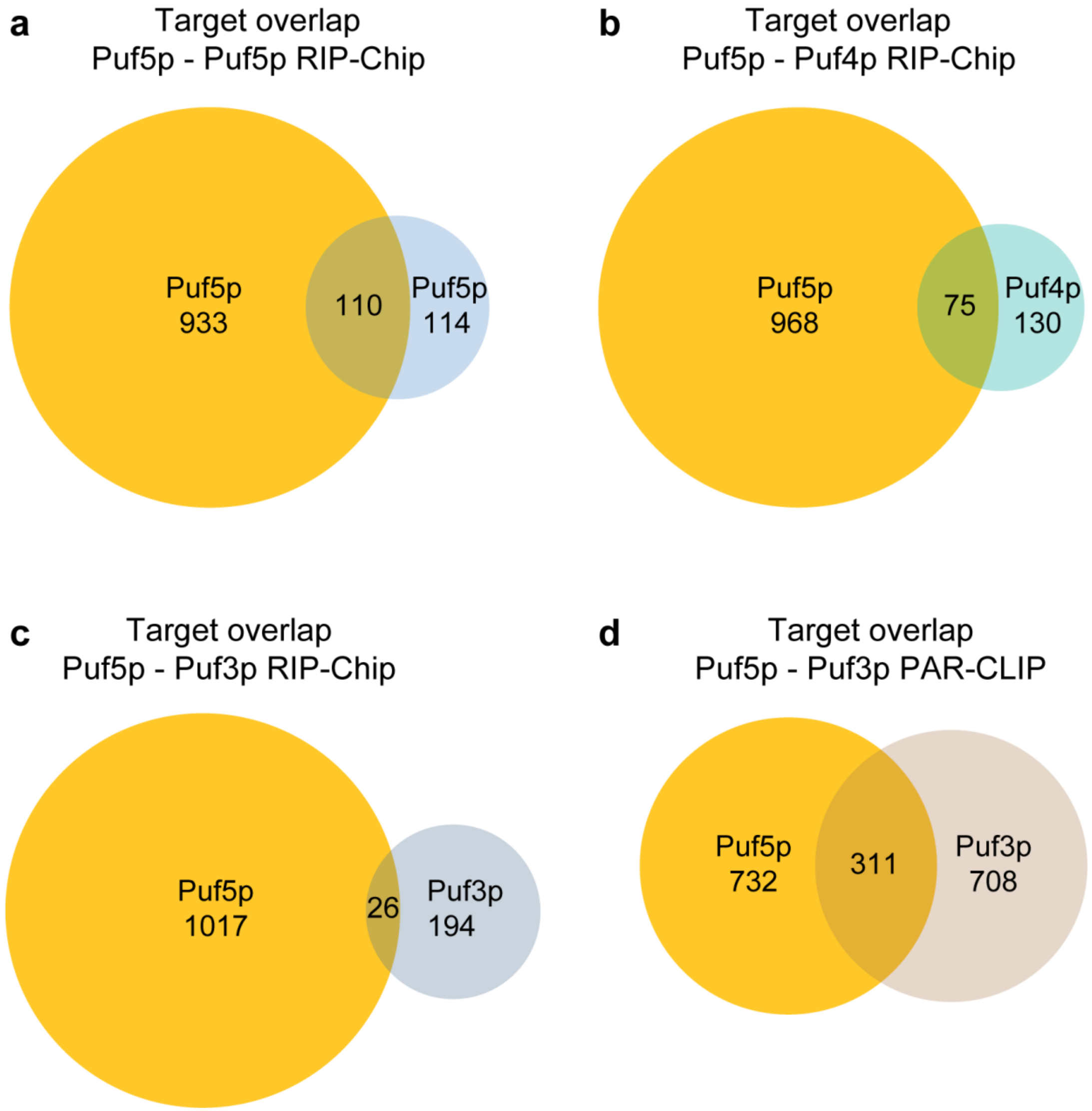

Supplementary Figure 3. Overlap of Puf5p HITS-CLIP targets with Puf3p and Puf4p targets. a. mRNA overlap between Puf5p HITS-CLIP and RIP-Chip[2]. b. mRNA overlap between Puf5p HITS-CLIP and Puf4p RIP-Chip[2]. c. mRNA overlap between Puf5p HITS-CLIP and Puf3p RIP-Chip[2]. d. mRNA overlap between Puf5p HITS-CLIP and Puf3p PAR-CLIP[3]. Numbers of mRNAs in each subset are indicated.

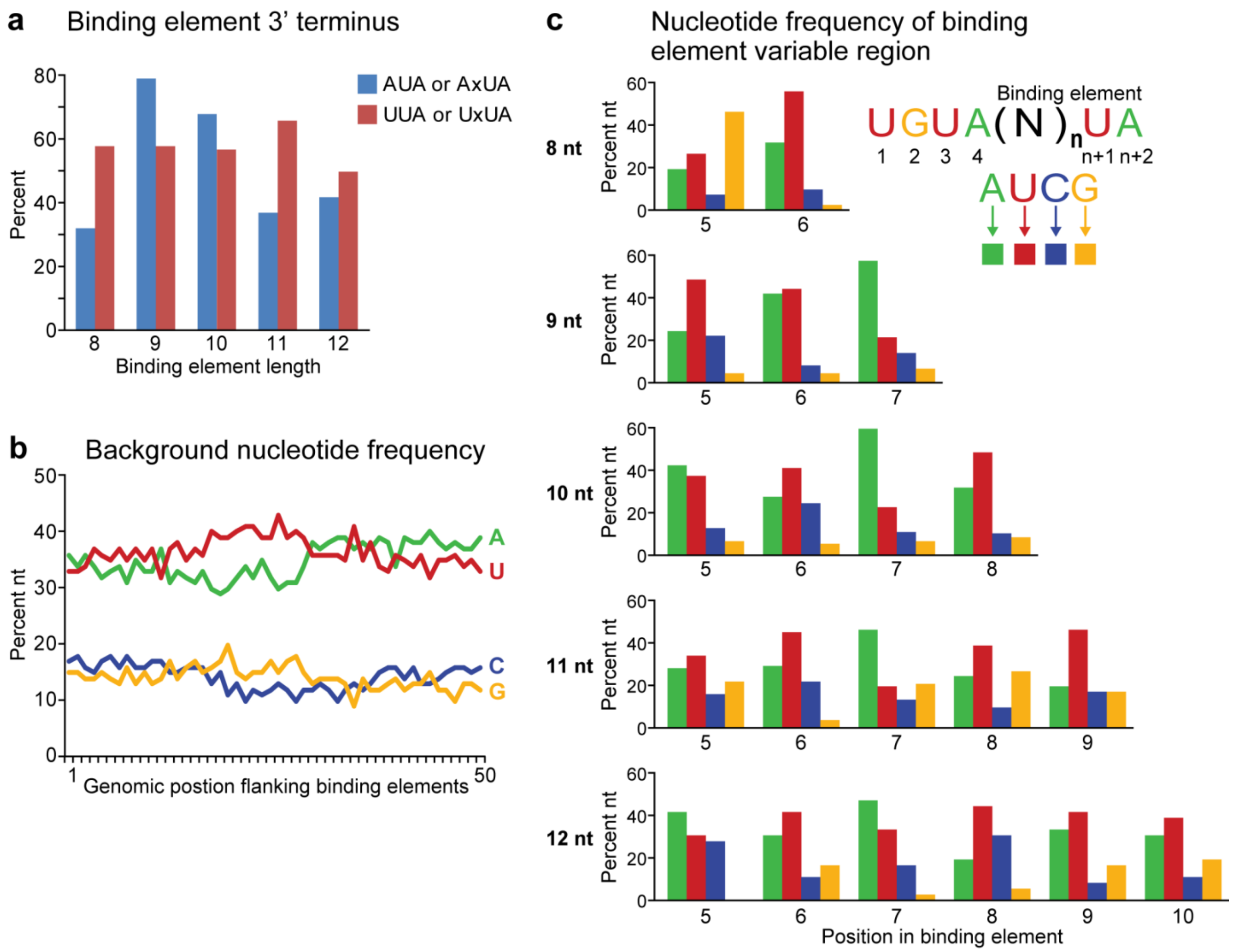

Supplementary Figure 4. Nucleotide composition of Puf5p binding elements. a. Enrichment for A's (blue), or U's (red) at the 3´ terminus of each length binding element. b. The frequency of each nucleotide flanking CLIP-defined peaks: A's (green), U's (red), C's (blue), and G's (orange). c. Positional analysis of intervening nucleotides (excluding the fixed 5´-UGUA tetranucleotide sequence and the 3´UA sequences) for each length binding element.

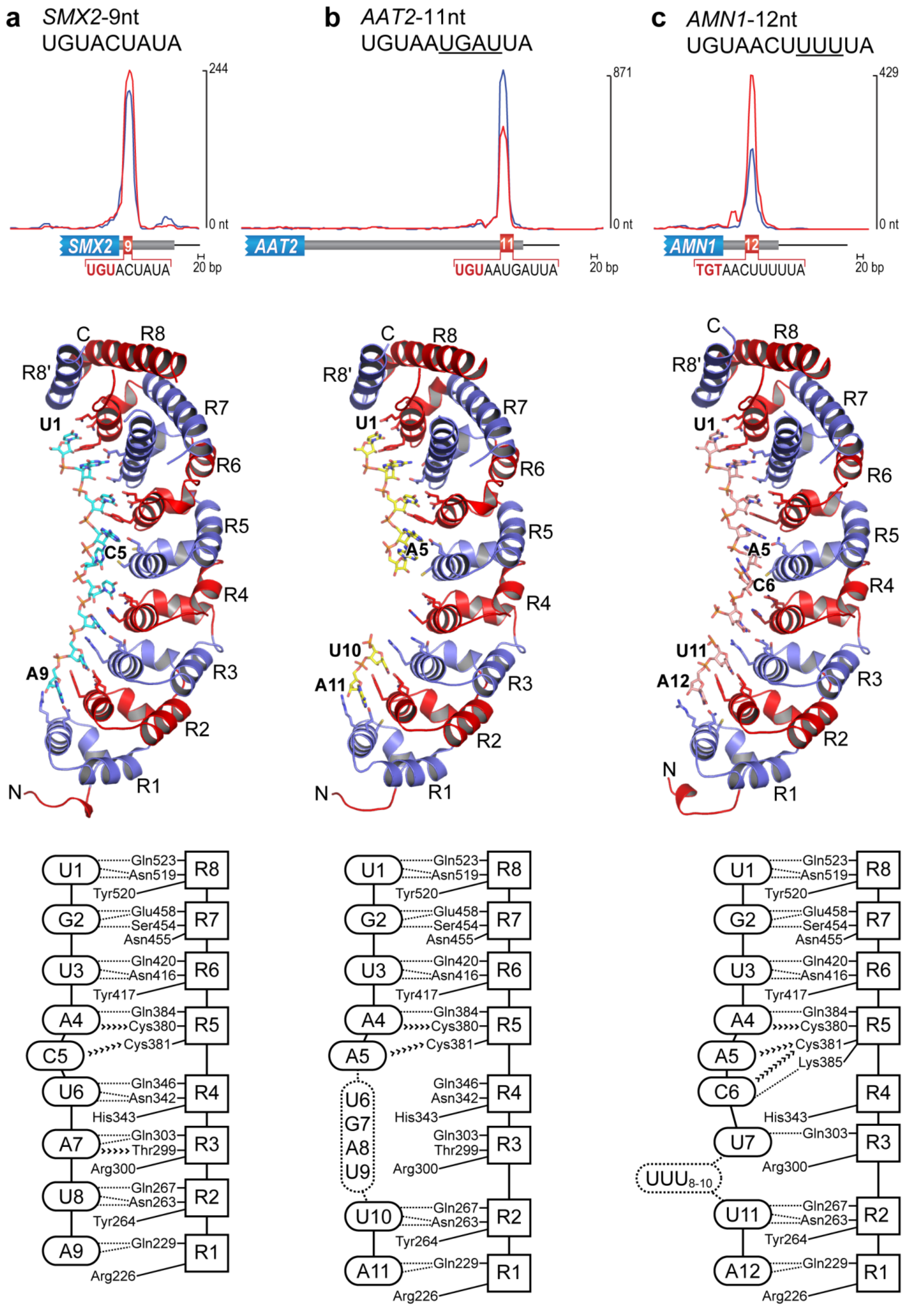

a
SMX2-9nt
UGUACUAUA
b
AAT2-11nt
UGUAAUGAUUA
c
AMN1-12nt
UGUAACUUUUUA
244
871
429
0 nt
SMX2
9
UGUACUAUA
AAT2
11
UGUAAUGAUUA
AMN1
12
TGTAACUUUUUA
20 bp
C
N
R1
R2
R3
R4
R5
R6
R7
R8
R8'
U1
C5
A9
A5
U10
A11
C6
U11
A12
G2
U3
A4
U6
A7
U8
G7
A8
U9
U7
UUU8-10
Gln523
Asn519
Tyr520
Glu458
Ser454
Asn455
Gln420
Asn416
Tyr417
Gln384
Cys380
Cys381
Lys385
Gln346
Asn342
His343
Gln303
Thr299
Arg300
Gln267
Asn263
Tyr264
Gln229
Arg226

Supplementary Figure 5. Crystal structures of Puf5p in complex with 9-nt *SMX2*, 11-nt *AAT2*, and 12-nt *AMN1* RNAs. HITS-CLIP peaks (a), ribbon drawings (b), and schematic diagrams of protein:RNA interactions (c) are shown.

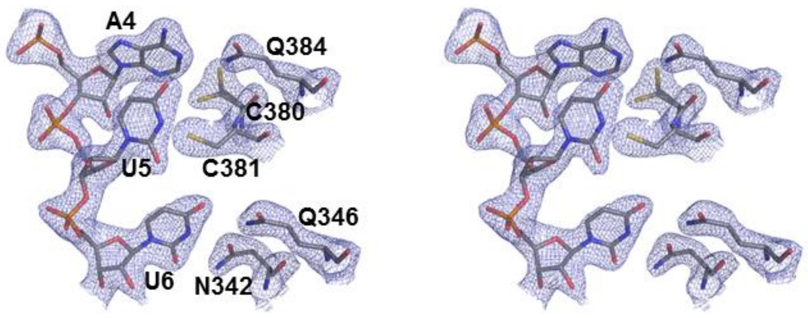

Supplementary Figure 6. Representative stereo image. Composite omit $2F_o$-F_c map, contoured at the 1.5 σ level, for the crystal structure of Puf5p in complex with 10-nt *MFA2* RNA. *MFA2* RNA bases 4-6 and RNA interacting residues from Puf5p are shown as stick models with atoms colored by element (carbon, grey; nitrogen, blue; oxygen, red; sulfur, yellow; phosphorus, orange). Cys380 adopts two alternative conformations.

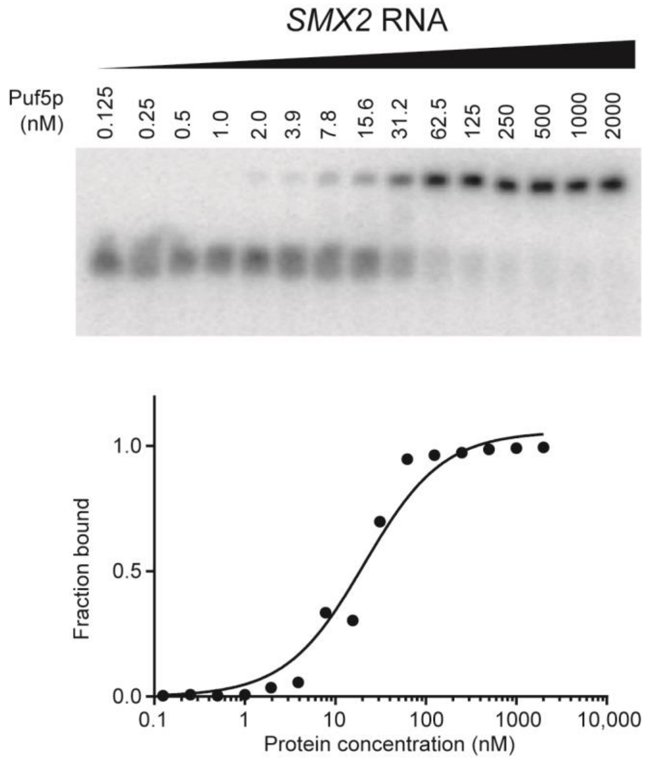

Supplementary Figure 7. Electrophoretic mobility shift assay (EMSA) of Puf5p. Representative EMSA of Pufp5 with *SMX2* binding element (UGUACUAUA) RNA (top) and data analysis (bottom) are shown. All binding assays were performed in triplicate and the mean K_d and standard error of the mean are reported in Supplementary Table 3.

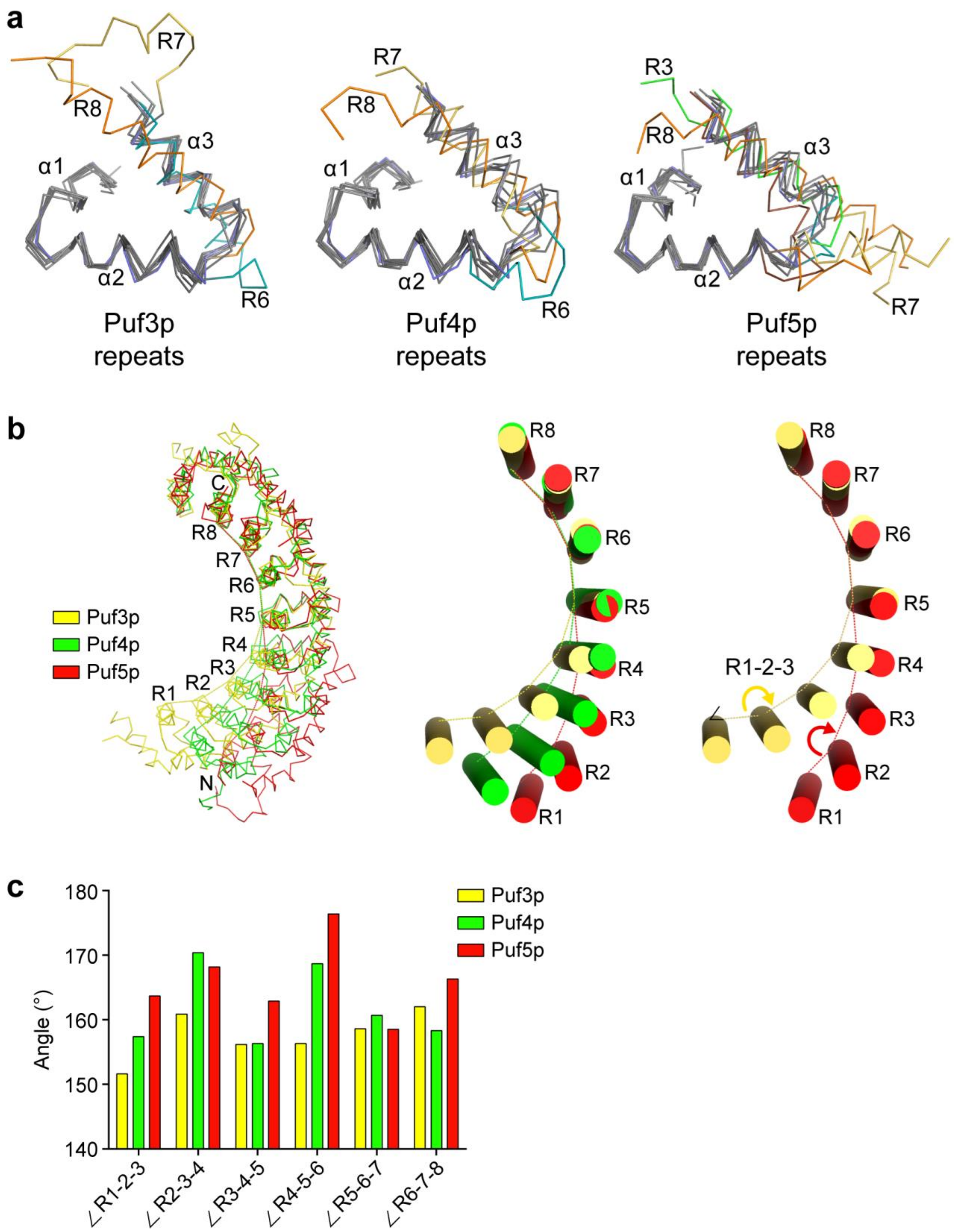

Supplementary Figure 8. Structural differences in yeast PUF proteins Puf3p, Puf4p, and Puf5p. a. Superposition of PUM repeats in Puf3p, Puf4p, and Puf5p. Cα traces of the eight repeats from each protein are superimposed with human PUM1 repeat 1 (blue). Divergent structures following helix α2 are colored teal (R6), yellow (R7), and orange (R8) for all proteins and green (R3) for Puf5p. b. Superposition of RNA-binding helices in Puf3p (yellow), Puf4p

(green) and Puf5p (red). Repeats 5-8 of each structure were aligned in the superposition. Cα traces are shown in the first, and the α2 helices only are shown as cylinders in the second and third set of traces. Dotted lines connecting the Cα atoms of base-stacking residues are shown, and ∠R1-2-3 is indicated . e. Repeat-to-repeat angles in Puf3p, Puf4p and Puf5p. The angles formed by lines between the Cα atoms of stacking residues in sets of three successive repeats for Puf3p, Puf4p and Puf5p are plotted.

a

Puf3 Repeat 6

```
                      Helix        Helix                   Helix
Scer|YLL013C         IPYLIQDQYGNYVIQYVLQQDQ---FTNKEMVDIKQE--IIETVANN
Smik|smik1210-g1.1   IPYLIQDQYGNYVIQYILQQNQ---FTNKEMVDVKQE--IIETVANN
Spar|spar52-g2.1     IPYLIQDQYGNYVIQYILQQDQ---FTNKEMVDIKQE--IIETVANN
Sbay|sbayc566-g3.1   IPYLIQDQYGNYVIQYILQQDQ---FTNKEMVDVKQE--IVETVADN
Ncas|Scas688.28      IPYLIQDQYGNYVIQHILEQQDNNPNVSQEMMNTKQE--IVNIVSQN
Sklu|SAKL0G13002g    IPYLIQDQYGNYVIQHILQHGG--EHTNIHIGSTKQN--IVDIVSKS
Cgla|CAGL0D05544g    IPYLIQDQYGNYVIQHILQHGSDVNLASEHMRVIKQE--IINNVADN
Egos|AAL152W         IPYLVQDQYGNYVIQHILQHGG-DNPAENHID--KSKQDIVDTISKT
Klac|KLLA0F15477g    IPFLIQDQYGNYVIQHILQHGT-EDTSSHIGMS-KQN--IIDIIRKN
Ylip|YALI0E12001g    AYHLIQDQYGNYVIQHVLEQGA---------PDDKEA--MMLVIKQH
Mgui|PGUG05371.1     IFYLIQDQYGNYVMQHILERGS---------SKDREA--ILEVVLGS
Ctro|CTRG03880.3     LYYLILDQYGNYVIQHILENGT---------PEEKEP--ILEIVLGS
Calb|orf19.1795      LYYLILDQYGNYVIQHILENGT---------QEEKEP--ILEIVLGS
Cpar|CPAG05281       LGFLITHKFGNYVIQACLENQ-----------LREQD--IFTTVVCK
Dhan|DEHA2C07128g    IFYLIQDQYGNYVMQHTLERGN---------PEDREE--ILKIVLGS
Clus|CLUG04502       LFYLIQDQYGNYVIQHILERGT---------PSEKEE--IFEVAFSS
Anid|AN6587          APRLIEDQYGNYVIQHIIQSGE---------EEDRSF--MIEMVKQK
Ncra|NCU06511        AHTLITDAYGNYVAQHIIEAGK---------PEDRAR--MIAAVMSQ
Sjap|SJAG00686       ILHLAQDQYGNYVIQHLMKKGS---------PSEQRE--IVEVVLGN
Soct|SOCG02114       SLQLTQGQYGNYVVQHILKEGS---------EKDKKF--VFNLIAKN
Spom|SPAC1687.22c    ILKLTQDQYGNYVVQHILRTGS---------ESDKKY--IFDLMIDH
```

b

Sc Puf3p

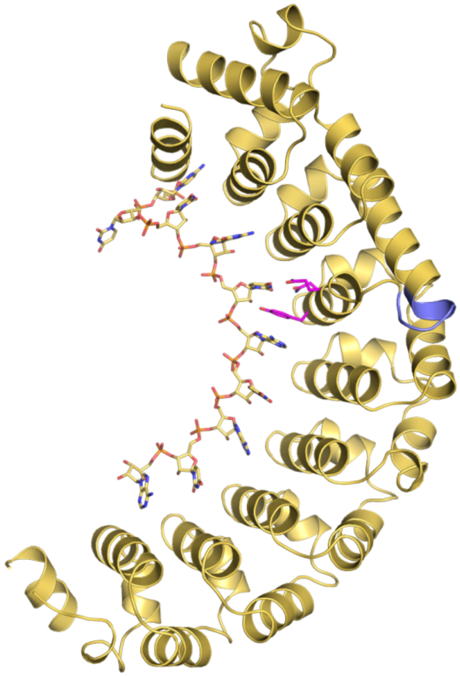

Supplementary Figure 9. Sequence analysis of Puf3p Repeat 6. a. Sequence alignment of Puf3p Repeat 6 from Ascomycota based on a MUSCLE multiple sequence aligment[4]. Positions of α helices in the crystal structure of *S. cerevisiae* Puf3p are indicated above the sequence alignment. Magenta highlighted residues represent the RNA-binding motif in *S. cerevisiae* Repeat 6 that is conserved in all species shown. The blue box indicates the additional residues at the interface of α helices 2 and 3 in Repeat 6. b. Ribbon drawing of the crystal structure of *S. cerevisiae* Puf3p in complex with a *COX17* binding element. RNA-binding motif residues of Repeat 6 are colored magenta and the additional loop residues are colored blue.

SUPPLEMENTARY TABLES

Supplementary Table 1 – Number of peaks containing binding elements

Single binding elements		Non-overlapping binding elements		Overlapping binding elements	
Binding element length (nt)	peaks	Binding element length (nt)	peaks	Binding element length (nt)	peaks
8	39	9,11	30	9-11	59
9	125	8,10	28	10-12	50
10	201	9,10	27	8-10	49
11	77	10,11	22	9-12	40
12	36	9,12	16	8-11	28
Total	478	10,12	15	8-12	28
		11,12	8	8-10-12	15
		8,11	7	8-10, 9-12	2
		9,11,12	7	8-10, 9-11	1
		8,9,10	6	8-11, 9-12	1
		9,10,11	6	8-12, 9-11	1
		8,10,11	5	8-11, 10-12	1
		8,10,12	4	Total	275
		8,12	4		
		8,9	4		
		8,9,10,12	4		
		9,10,12	4		
		10,11,12	2		
		8,9,11	2		
		Total	201		
				Total	954

Supplementary Table 1. Number of peaks containing binding elements. Left columns show the numbers of peaks posses only one binding element. The middle and right columns contain the number of peaks that posses non-overlapping or overlapping binding elements, respectively.

Supplementary Table 2 – Significantly enriched Gene Ontology terms for each binding element length and all targets.

**Binding element length**	**Enriched GO term**	**p-value (Hypergeometric distribution test Holm-Bonferroni corrected)**
**8**	154 genes used only UGUA set (all 8's)	
	**mitochondrion organization**	**9.50E-04**
	protein targeting to mitochondrion	3.90E-03
	mitochondrial transmembrane transport	5.82E-03
	protein localization to mitochondrion	1.14E-02
	establishment of protein localization to mitochondrion	1.14E-02
	protein import into mitochondrial matrix	2.03E-02
**9**	250 genes total only UGUA set (all 9's)	
	ribonucleoprotein complex biogenesis	2.83E-15
	**ribosome biogenesis**	**3.59E-14**
	ncRNA metabolic process	1.76E-06
	rRNA processing	1.79E-06
	rRNA metabolic process	2.18E-06
	cellular component biogenesis	1.21E-05
	ncRNA processing	1.69E-05
	RNA metabolic process	1.50E-04
	RNA processing	4.15E-04
	ribosomal large subunit biogenesis	5.72E-04
	nucleocytoplasmic transport	1.19E-03
	nuclear transport	1.33E-03
	maturation of 5.8S rRNA	2.45E-03
	maturation of 5.8S rRNA from tricistronic rRNA transcript (SSU-rRNA, 5.8S rRNA, LSU-rRNA)	2.45E-03
	maturation of LSU-rRNA	4.42E-03
	nucleic acid metabolic process	5.53E-03
	nuclear export	6.22E-03
	nucleobase-containing compound metabolic process	6.43E-03
	gene expression	6.82E-03
	maturation of LSU-rRNA from tricistronic rRNA transcript (SSU-rRNA, 5.8S rRNA, LSU-rRNA)	7.75E-03
	ribosomal small subunit biogenesis	1.80E-02
	cellular component organization or biogenesis	2.94E-02
	cellular aromatic compound metabolic process	3.66E-02
**10**	353 genes total only UGUA set (all 10's)	

	regulation of macromolecule biosynthetic process	3.03E-07
	regulation of biosynthetic process	3.48E-07
	regulation of cellular macromolecule biosynthetic process	4.97E-07
	regulation of cellular biosynthetic process	7.85E-07
	regulation of macromolecule metabolic process	1.80E-06
	regulation of primary metabolic process	2.54E-06
	**regulation of gene expression**	**2.85E-06**
	transcription, DNA-templated	3.64E-06
	nucleic acid-templated transcription	3.89E-06
	RNA biosynthetic process	5.41E-06
	regulation of cellular metabolic process	7.37E-06
	regulation of metabolic process	1.46E-05
	regulation of cellular process	1.66E-04
	regulation of biological process	1.69E-04
	biological regulation	2.83E-04
	regulation of nucleobase-containing compound metabolic process	5.97E-04
	regulation of nitrogen compound metabolic process	6.05E-04
	regulation of transcription, DNA-templated	8.28E-04
	regulation of nucleic acid-templated transcription	8.78E-04
	regulation of RNA biosynthetic process	8.78E-04
	RNA metabolic process	9.58E-04
	regulation of RNA metabolic process	1.07E-03
	nucleobase-containing compound biosynthetic process	1.54E-03
	organic cyclic compound biosynthetic process	1.69E-03
	transcription from RNA polymerase II promoter	1.90E-03
	aromatic compound biosynthetic process	2.59E-03
	heterocycle biosynthetic process	3.31E-03
	**chromatin modification**	4.66E-03
	cellular macromolecule biosynthetic process	6.60E-03
	cellular nitrogen compound biosynthetic process	7.26E-03
	chromatin organization	1.04E-02
	macromolecule biosynthetic process	1.04E-02
	**chromatin remodeling**	2.16E-02
	gene expression	2.47E-02
	nucleic acid metabolic process	3.56E-02
	cellular biosynthetic process	4.27E-02
	nucleobase-containing compound metabolic process	4.66E-02
	cellular aromatic compound metabolic process	4.75E-02

**11**	185 genes total only UGUA set (all 11's)	
	**translation**	**3.60E-03**
	gene expression	4.12E-03
	cytoplasmic translation	1.73E-02
**12**	169 genes total only UGUA set (all 11's)	
	none	
**All**	1043 genes total	
	**cytoplasmic translation**	**8.04E-52**
	ribonucleoprotein complex biogenesis	6.25E-11
	**ribosome biogenesis**	**2.44E-10**
	organic substance biosynthetic process	3.52E-10
	biosynthetic process	3.37E-09
	cellular biosynthetic process	3.30E-08
	gene expression	5.06E-08
	cellular macromolecule biosynthetic process	9.34E-08
	macromolecule biosynthetic process	1.86E-07
	**translation**	**1.83E-06**
	nucleic acid transport	4.84E-06
	nucleobase-containing compound transport	6.56E-06
	ribosomal small subunit biogenesis	7.98E-06
	RNA transport	8.37E-06
	establishment of RNA localization	8.37E-06
	ribosome assembly	1.68E-05
	ncRNA metabolic process	2.49E-05
	RNA localization	3.52E-05
	ribonucleoprotein complex assembly	6.73E-05
	nuclear transport	8.92E-05
	ribonucleoprotein complex subunit organization	1.15E-04
	rRNA export from nucleus	1.51E-04
	rRNA transport	1.51E-04
	nucleocytoplasmic transport	1.84E-04
	transcription, DNA-templated	2.29E-04
	nucleic acid-templated transcription	2.54E-04
	RNA biosynthetic process	2.55E-04
	cellular component organization or biogenesis	3.39E-04
	ribonucleoprotein complex export from nucleus	4.14E-04
	regulation of gene expression	4.19E-04
	cellular component biogenesis	6.49E-04
	maturation of SSU-rRNA	6.63E-04

	ribosomal large subunit biogenesis	6.64E-04
	ribonucleoprotein complex localization	7.30E-04
	regulation of cellular macromolecule biosynthetic process	7.46E-04
	nuclear export	7.57E-04
	regulation of macromolecule biosynthetic process	8.16E-04
	macromolecular complex subunit organization	1.04E-03
	regulation of biosynthetic process	1.83E-03
	**chromatin remodeling**	**1.98E-03**
	regulation of cellular biosynthetic process	2.65E-03
	ribosomal large subunit assembly	3.50E-03
	maturation of SSU-rRNA from tricistronic rRNA transcript (SSU-rRNA, 5.8S rRNA, LSU-rRNA)	4.02E-03
	RNA export from nucleus	4.18E-03
	**chromatin organization**	**4.36E-03**
	transcription from RNA polymerase I promoter	4.67E-03
	**chromatin modification**	**5.09E-03**
	RNA metabolic process	5.21E-03
	regulation of macromolecule metabolic process	5.31E-03
	regulation of translation	5.87E-03
	posttranscriptional regulation of gene expression	7.42E-03
	ncRNA transcription	7.99E-03
	pyruvate metabolic process	8.07E-03
	nucleobase-containing compound biosynthetic process	8.31E-03
	regulation of primary metabolic process	1.35E-02
	rRNA-containing ribonucleoprotein complex export from nucleus	1.45E-02
	rRNA processing	1.51E-02
	organic cyclic compound biosynthetic process	1.54E-02
	rRNA metabolic process	1.99E-02
	nucleobase-containing compound metabolic process	2.08E-02
	nucleobase-containing small molecule metabolic process	2.57E-02
	regulation of translational elongation	3.48E-02
	organelle assembly	3.96E-02
	regulation of cellular metabolic process	4.79E-02
	cytosolic ribosome	2.25E-54
	cytosolic part	6.05E-53
	ribosomal subunit	8.61E-35
	ribosome	1.24E-34
	cytosolic large ribosomal subunit	4.10E-30

	ribonucleoprotein complex	3.73E-24
	cytosolic small ribosomal subunit	7.12E-24
	macromolecular complex	1.04E-18
	non-membrane-bounded organelle	2.09E-17
	intracellular non-membrane-bounded organelle	2.09E-17
	large ribosomal subunit	1.86E-16
	small ribosomal subunit	3.87E-16
	preribosome	3.08E-12
	cytosol	3.81E-07
	intracellular organelle	3.08E-06
	preribosome, large subunit precursor	6.29E-06
	organelle	6.73E-06
	extracellular region	1.27E-05
	cell	2.44E-05
	cell part	2.44E-05
	90S preribosome	1.85E-04
	**fungal-type cell wall**	**5.47E-04**
	intracellular organelle part	1.30E-03
	**cell wall**	**1.31E-03**
	external encapsulating structure	1.31E-03
	organelle part	1.84E-03
	nuclear nucleosome	5.06E-03
	DNA-directed RNA polymerase I complex	9.83E-03
	**SWI/SNF superfamily-type complex**	**1.00E-02**
	nucleosome	1.50E-02
	DNA-directed RNA polymerase complex	2.73E-02
	RNA polymerase complex	2.73E-02
	cytoplasmic part	3.66E-02
	nuclear DNA-directed RNA polymerase complex	4.48E-02

Supplementary Table 2. GO terms enrichments. GO terms for all Puf5p targets and those with specific length binding elements.

Supplementary Table 3 - Puf5p Electrophoretic Mobility Shift Assays

RNA	RNA sequence	K_d (nM)	K_{rel}
NRE (8)	UGUAUAUA	3200 ± 270	113
SMX2 (9)	UGUACUAUA	28.4 ± 3.8	1
MFA2 (10)	UGUAUUUGUA	179.4 ± 38.3	6.3
AAT2 (11)	UGUAAUGAUUA	345.2 ± 61.4	12.2
AMN1 (12)	UGUAACUUUUUA	106.0 ± 9.7	3.7

EMSAs were performed in triplicate and the mean K_d ± standard error of the mean are reported as well as K_d's relative to binding to the 9 nt *SMX2* RNA, which was set to K_{rel}=1.

Supplementary Table 3. Puf5p Electrophoretic Mobility Shift Assays (EMSAs). EMSAs were performed in triplicate and the mean K_d ± standard error of the mean are reported as well as K_d's relative to binding to the 9 nt *SMX2* RNA, which was set to K_{rel}=1.
